# Two- and three-dimensional neuropeptidomic landscape in the central nervous system of an invertebrate chordate, *Ciona robusta*

**DOI:** 10.1016/j.isci.2025.113413

**Published:** 2025-08-21

**Authors:** Tomohiro Osugi, Akira Shiraishi, Yasunori Sasakura, Kohtaro Sugahara, Tohru Yamagaki, Tatsuya Yamamoto, Honoo Satake

**Affiliations:** 1 Bioorganic Research Institute, Suntory Foundation for Life Sciences, Soraku-gun, Kyoto 619-0284, Japan; 2 Shimoda Marine Research Center, University of Tsukuba, Shimoda, Shizuoka 415-0025, Japan

**Keywords:** Molecular biology, Neuroscience, Evolutionary biology, Omics

## Abstract

Neuroanatomical information on neuropeptidergic neurons provides crucial clues to understanding the functions of neuropeptides and the cerebral structure-function relationships. In this study, we constructed comprehensive two- and three-dimensional atlases of 23 neuropeptides in the central nervous system (CNS) of an invertebrate chordate, *Ciona robusta*, at the single-cell level using a mass spectrometry imaging workflow. The neuropeptidomic atlases revealed that the cerebral ganglion is divided into three functional areas, dorso-rostral, dorso-caudal, and ventral areas. Particularly, the distribution of *Ciona* tachykinin, cholecystokinin, and neurotensin-like-peptide demonstrated the basal cerebral structure–function relationships in reproductive regulation in *Ciona*. The atlases also demonstrated the unexpected colocalization of multiple neuropeptides and the differential localization of gene-related peptides yielded from a single precursor, which cannot be unraveled by any transcriptomic analyses. Collectively, the present study provides a foundation for investigating various neuropeptidergic functions of the invertebrate chordate CNS and the evolution of chordate neuropeptidergic systems.

## Highlights

• An MSI workflow created 2D and 3D neuropeptidomic atlases of *Ciona* brain
• Atlases showed unexpected peptide localization and their differences
• Atlases showed structure-function relationships related to reproductive regulation
• Atlases provide insights into the evolution of chordate neuropeptidergic systems

## Graphical abstract

**Figure:**
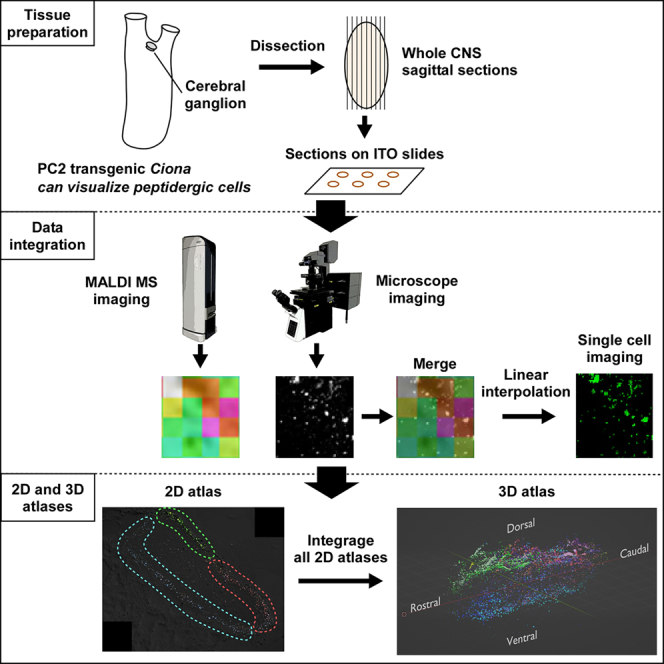

## Introduction

Neuropeptides and peptide hormones, which are produced in various organs, including the nervous and endocrine systems, are essential regulatory factors for various biological processes in the animal kingdom. The central nervous system (CNS) plays major roles in producing neuropeptides and possesses common and unique structural and functional characteristics among animal species. Thus, the in-depth elucidation of the neuropeptidergic structure–function relationships of CNSs is expected to provide crucial clues for understanding the evolutionary lineages of metazoan CNSs as well as the neuropeptidergic system of an organism.

*Ciona intestinalis* type A (synonym of *Ciona robusta*) is a cosmopolitan species among ascidians and is the closest living relative to vertebrates.^1^^,^^2^ Thus, investigations of the *Ciona* CNS at the anatomical and molecular levels will lead not only to the understanding of the structure‒function relationships of the *Ciona* CNS but also to significant insights into the origin of those of chordate brains.^3^^,^^4^ *Ciona* larva has been studied on the CNS system with several neurotransmittergic and neuropeptidergic networks,^5^^,^^6^^,^^7^ which regulate the sensory and motor functions, and metamorphosis of larva.^7^^,^^8^^,^^9^^,^^10^ However, the larval CNS is rearranged during metamorphosis, and major organs, including digestive, cardiovascular, muscular, and reproductive organs, develop after metamorphosis.^11^ Therefore, the adult *Ciona* CNS provides attractive targets for the investigation of the regulatory systems involved in various basal biological actions, such as reproduction and food intake, as well as their evolutionary aspects of chordate CNSs. The CNS of the adult *Ciona*, called the neural complex, consists of the cerebral ganglion and neural gland, and the cerebral ganglion is a counterpart of the brain in vertebrates.^1^ In contrast to those in the larvae, the structure‒function relationships of the cerebral ganglion in *Ciona* adults largely remain to be understood. Nevertheless, over the past decade, we have identified more than 30 neuropeptides, including both orthologs of vertebrate peptides and various *Ciona*-specific peptides from the neural complex of *Ciona,* by peptidomics.^6^^,^^12^^,^^13^ Furthermore, we verified the biological roles of the identified neuropeptides in the ovary; *Ciona* tachykinin (CiTK),^6^^,^^12^^,^^13^^,^^14^^,^^15^ *Ciona* neurotensin-like peptide-6 (CiNTLP-6),^6^^,^^12^^,^^13^ *Ciona* vasopressin (CiVP),^6^^,^^13^^,^^16^^,^^17^ and cionin (*Ciona* cholecystokinin)^18^ regulate follicle growth,^6^^,^^12^^,^^13^^,^^14^^,^^15^ maturation,^6^^,^^13^^,^^16^^,^^17^ and ovulation.^6^^,^^13^^,^^16^^,^^17^^,^^18^ These findings indicate that neuropeptides play central roles in the regulation of biological processes via the neural complex in *Ciona*.

Localization, namely, the visualization of neuropeptides by immunostaining, provides fundamentals for investigating the structure‒function relationships of CNSs. However, immunostaining has critical disadvantages in the localization of multiple peptides. First, antibodies against peptides frequently exhibit cross-reactivity, which hampers the rigorous detection of structurally related peptides (peptide subtypes). Second, current immunostaining methods (e.g., fluorescent or chemiluminescent detection) can detect at most three different peptides in a single section in principle and thus cannot simultaneously localize numerous peptides characterized by omics analyses. In addition, current spatial transcriptomes, including single-cell RNA-seq, provide incomplete information on mature peptide localization, given that multiple gene-related peptides are generated from a transcript encoding a single precursor and that some of them are differentially distributed in different cells without the generation of splicing variants.^19^

The technological advances over the past decades in mass spectrometry (MS) enabled single cell and subcellular imaging analyses using various methods, including MALDI-TOF MS, secondary ion mass spectrometry (SIMS), capillary electrophoresis mass spectrometry (CE-MS), or multiplexed ion beam imaging by time-of-flight (MIBI-TOF).^20^ Among these, MALDI-TOF MS is particularly versatile and well-suited for label-free and non-targeted analyses, and has provided valuable insights into the distribution of biomolecules and their metabolites.^20^^,^^21^^,^^22^^,^^23^^,^^24^^,^^25^ The sensitivity of MALDI-TOF MS was shown to be sufficient for the detection of neuropeptides from isolated single cells.^25^^,^^26^^,^^27^^,^^28^^,^^29^ In addition to these works, the fluorescence cell labeling was applied to isolate and analyze specific neurons using MS in the transgenic *Drosophila*.^30^ These foundational studies suggest that MALDI-TOF MS is suitable for single cell analysis on intact tissue sections. Mass spectrometry imaging (MSI) is a powerful tool for simultaneously detecting and visualizing numerous peptides within a single tissue section. MSI provides two-dimensional (2D) images of the spatial distribution of biomolecules, including neuropeptides, in both invertebrates and vertebrates.^31^^,^^32^ Moreover, three-dimensional (3D) MSI have been developed in earlier studies, enabling visualization of biological molecules in animal organs at spatial resolutions of 100–200 μm.^33^^,^^34^ More recently, 3D MSI was further advanced to visualize the molecular distributions in the kidney at a resolution of 50 μm. These studies demonstrate that 2D and 3D MSI have great potential for revealing the spatiotemporal distribution of biomolecules in various organs, although single-cell 3D MSI remains challenging. While 3D single-cell MSI has been successfully applied to map lipid distributions in fertilized zebrafish embryos at the one-cell stage, where cell size exceeds 600 μm in diameter,^35^ it is still difficult to apply this technique to organs containing small neurons less than 20 μm in diameter.

In this study, we constructed 2D and 3D atlases for the localization of 23 neuropeptides in the *Ciona* cerebral ganglion at the single-cell (∼20 μm) level using optimized MSI analysis of transgenic *Ciona* adults in which neuropeptidergic neurons are visualized.^36^^,^^37^ These “neuropeptidomic landscapes” showed unexpected colocalization combinations of multiple peptides in peptidergic neurons that could not be identified by any current single-cell RNA-seq or conventional histological methods, and provided crucial clues for investigating cerebral structure–function relationships of the invertebrate chordate. Overall, the present study paves the way for understanding the evolutionary origin of neuropeptidergic systems in chordate CNSs and highlights the usefulness and versatility of the present single-cell MSI model for elucidating the diverse neuropeptide landscapes.

## Results

### Mass spectrometry imaging of the *Ciona* neuropeptides

**Figure 1:**
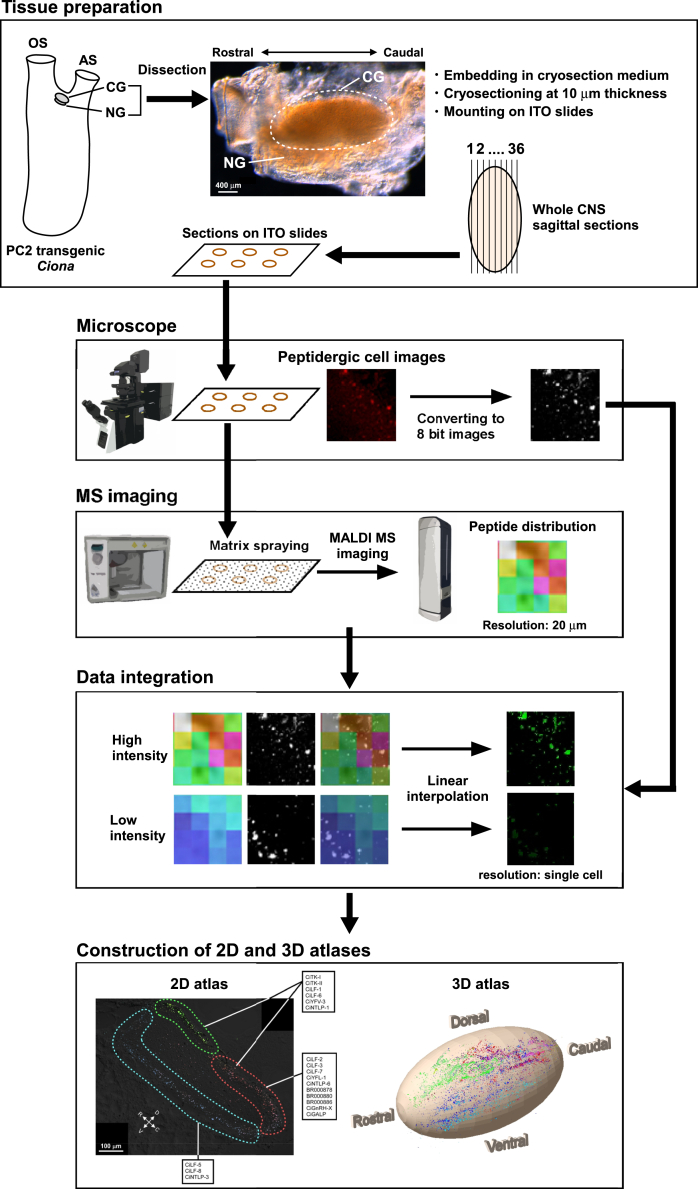
Workflow for the construction of 2D and 3D peptide atlases of the *Ciona* cerebral ganglion *Tissue preparation*. The *Ciona* central nervous system (CNS) consists of the cerebral ganglion (CG), which is a neural tissue, and the neural gland (NG), which is a nonneural glandular tissue. The CG is indicated by a dotted line. Tissues were dissected from PC2 transgenic *Ciona*, in which peptidergic cells were labeled with the Kaede fluorescent protein. Whole CNS sagittal sections were mounted on ITO slides. *Microscope and MS imaging*. Images of the peptidergic cells were acquired with a confocal microscope. The sections were then subjected to matrix spraying and MALDI MS imaging. *Data integration*. Peptidergic cell fluorescence confocal microscopy images and MS images of peptide distribution were integrated as mapping data using the linear interpolation method with the data processing program. *Construction of 2D and 3D atlases*. Single-cell-level peptide localization was visualized using the mapping data of all sections, and 2D and 3D atlases were constructed with graphic software (GIMP for 2D and blender for 3D). OS, oral siphon; AS, atrial siphon. See Figure S1 for the data processing program.

**Figure 2:**
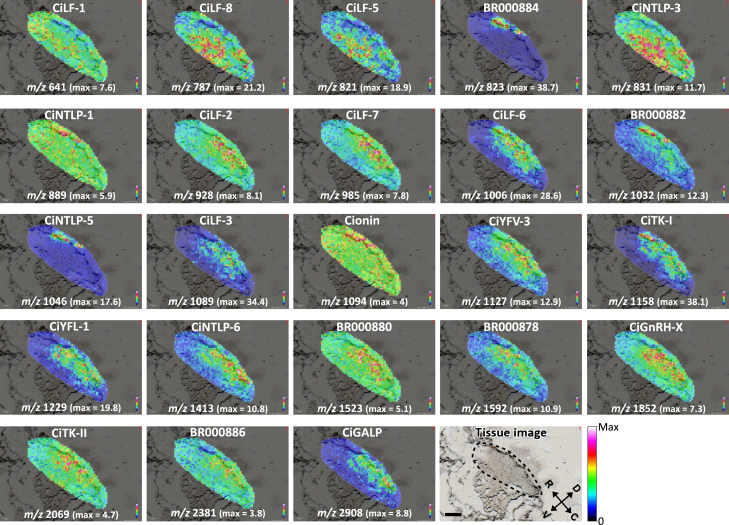
MSI of *Ciona* peptides in a single section of the cerebral ganglion The images show the distribution of the respective *Ciona* peptide in a single sagittal section of the cerebral ganglion. In each image, the detected peptides are shown as 20 × 20-μm pixels. The colors of the pixels indicate the relative intensities of the detected peptides. The names, mass values, and max intensities of each peptide are shown in the images. The color bar is shown at the bottom right. The orientation of the section is indicated by arrows in the tissue image, and the cerebral ganglion is indicated by a dotted line. R, rostral; C, caudal; D, dorsal; V, ventral. Bar, 200 μm. See Figure S3 for MS/MS spectra of each peptide.

The workflow of the present study is summarized in Figure 1. We first performed normal MSI on sections of the *Ciona* cerebral ganglion to confirm the presence of *Ciona* peptides before the construction of 2D and 3D peptide atlases. The cerebral ganglion is a simple oval-shaped neural tissue (Figure 1).^1^ Most of the neurons are distributed in the outer area of the cerebral ganglion, and nerve fibers accumulate in the inner area.^1^ To date, more than 30 peptides have been identified in the *Ciona* cerebral ganglion by our previous studies, including MS-based peptidomic analysis.^12^ Based on the peptidomic data, MSI analysis was performed on *Ciona* cerebral ganglion sections in this study. MSI simultaneously detected the parent masses of the 23 neuropeptides and revealed their distribution in cerebral ganglion sections at a spatial resolution of 20 μm, demonstrating the specific localization of each peptide (Figure 2). The names and mass values of the peptides detected by MSI are summarized in Table S1. To confirm the peptide sequences detected by MSI, MS/MS analysis of the peptides was performed on cerebral ganglion sections. The fragment ions, such as b-ions and y-ions, of most peptides in tissue sections coincided with those of synthetic peptides (Figure S3). The average mass error of the fragment ions was approximately 260 ppm, confirming that the fragment ions were accurately detected (Figure S3).^38^ These results provide evidence that *Ciona* neuropeptides were detected via MSI in cerebral sections.

### Image processing of the mass spectrometry imaging data

**Figure 3:**
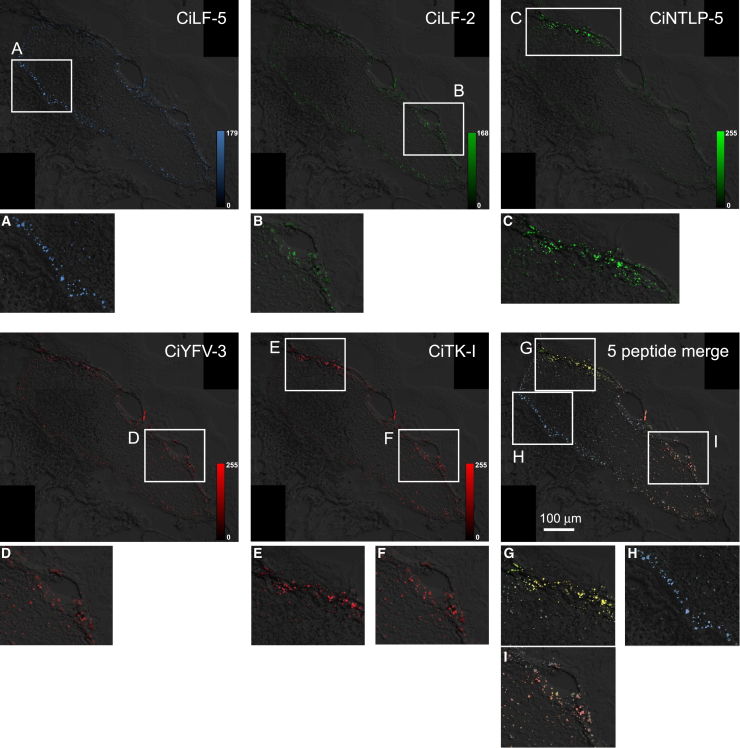
Image processing of the MSI data The distributions of five peptides (CiLF-5, blue; CiLF-2, green; CiNTLP-5, green; CiYFV-3, red; and CiTK-I, red) are shown in a single section of the cerebral ganglion. The rectangular areas where the peptides are mainly localized (A–F) are magnified below. The merged image is shown at the bottom right. See Figure S2A for the distribution of the peptidergic cells. See Figure S4 for the distribution of each peptide. Bar, 100 μm.

The resolution of the MSI image is 20 μm, which is too large to identify the respective peptidergic neurons at the level of single cells as the size of neurons in the *Ciona* cerebral ganglion ranges from 5 to 14 μm (Figure 1).^1^ To verify the distribution of peptidergic cells, we initially integrated MSI data and fluorescence microscopy images of cerebral ganglion sections from proprotein/prohormone convertase 2 (PC2) promoter-Kaede transgenic *Ciona* in which almost all peptidergic neurons were indicated by Kaede-derived fluorescence (Figures 1, S1, and S2A).^36^^,^^37^ After the integration of these data by linear interpolation, cells with specific MS data were labeled with pseudocolors as the mapping images (Figure 1). These data led to the visualization of the distribution of 23 neuropeptide signals in cerebral ganglion sections at the level of single cells (Figure S4). Among the 23 peptides, any mapping data of interest can be compared or merged on demand. Figure 3 shows an example of a comparison of the distribution of five peptide signals (e.g., CiLF-2, CiLF-5, CiNTLP-5, CiYFV-3, and CiTK-I) in a single section. These MSI analyses demonstrated both differential localization and colocalization of these five *Ciona* neuropeptides in the cerebral ganglion (Figure 3).

**Figure 4:**
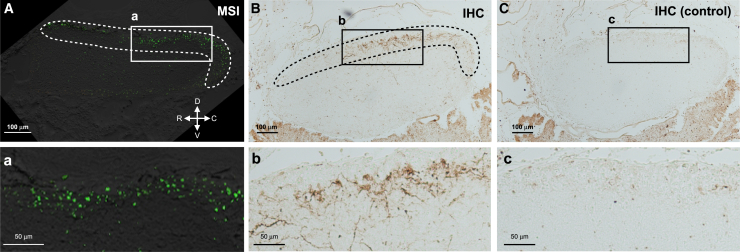
Comparison of the distribution of CiTK-I between the MSI and immunohistochemistry images MSI of CiTK-I (A) and immunohistochemistry using antiserum against CiTK-I (B). The areas where CiTK-I is mainly distributed are indicated by dotted lines. The rectangular areas are magnified below (a–c). All the immunoreactivities disappeared in the control group (C). The orientation of the tissue is indicated by arrows. R, rostral; C, caudal; D, dorsal; V, ventral. See Figure S2B for the specificity of the antiserum against CiTK-I. Bars, 100 μm (A–C), 50 μm (a–c).

To confirm the reliability of peptide localization visualized by the present MSI analyses, we compared the results of MSI and immunohistochemistry for several peptides. MSI showed that CiTK-I was mainly distributed in the dorsal area of the cerebral ganglion (Figure 4). Immunohistochemistry using antiserum against CiTK-I, which specifically reacts with CiTK-I (Figure S2B), also revealed immunoreactive cells and fibers in the same area (Figure 4). Interestingly, MSI showed differential localization of CiLF peptide signals including CiLF-2, CiLF-5, CiLF-6, and CiLF-7, which are produced from a single precursor protein (Figure S5Aa).^12^ CiLF-6 was distributed in the dorso-rostral area (Figure S5Aa’), CiLF-5 was distributed in the ventro-rostral area (Figure S5Ab’), and CiLF-2 and CiLF-7 were distributed in the dorso-caudal area (Figure S5Ac’). These MSI analyses suggest that CiLFs, which are encoded by a single precursor without any splicing variants,^12^ were differentially produced among neurons. Immunohistochemistry using the antiserum against CiLF-2, which exhibited cross-reactivity to CiLF-2, CiLF-5, CiLF-6, and CiLF-7 (Figure S2Bb), showed immunoreactive signals in all areas where MSI showed the distribution of CiLF-2, CiLF-5, CiLF-6, and CiLF-7, respectively (Figure S5Ad’-S5Af’). These immunoreactive signals disappeared after preabsorbing CiLF-2, CiLF-5, CiLF-6, and CiLF-7 (Figure S5Ag’–S5Ai’). These results are in good agreement with the cross-reactivity of the CiLF-2 antibody against the CiLF peptides and corroborate the accuracy of the MSI analysis (Figure S2Bb).

We further compared the localization of neuropeptides by MSI with the spatial gene expression mapping system, Visium (Figure S5B). The distributions of CiTK-I and *citk* mRNA, and of CiNTLP-3 and *cint-A* mRNA were similar in some regions, where numerous peptidergic neurons reside (Figures S5Ba and S5Bb). Furthermore, as mentioned above, CiLF-2, CiLF-5, CiLF-6, and CiLF-7, which are encoded by a single gene, *cilf*, exhibited different distributions in the cerebral ganglion according to MSI (Figure S5Bc), whereas the distribution of *cilf* mRNA roughly coincided with that of the corresponding CiLF peptides (Figure S5Bc). However, the distributions of CiTK-I, CiNTLP-1, and CiLFs in the inner area of the cerebral ganglion, where only a small number of peptidergic neurons are present, were evidently greater in the Visium group than in the MSI group, indicating the limitation of Visium in analyses of small organs, including the *Ciona* cerebral ganglion. (Figures S5Ba–S5Bc). Collectively, these data showed that the present MSI analysis provides much more accurate and informative data for the localization of multiple *Ciona* neuropeptides at the single-cell level than does the current on-tissue transcriptome analysis.

### Neuropeptidomic atlas construction of the *Ciona* cerebral ganglion

**Figure 5:**
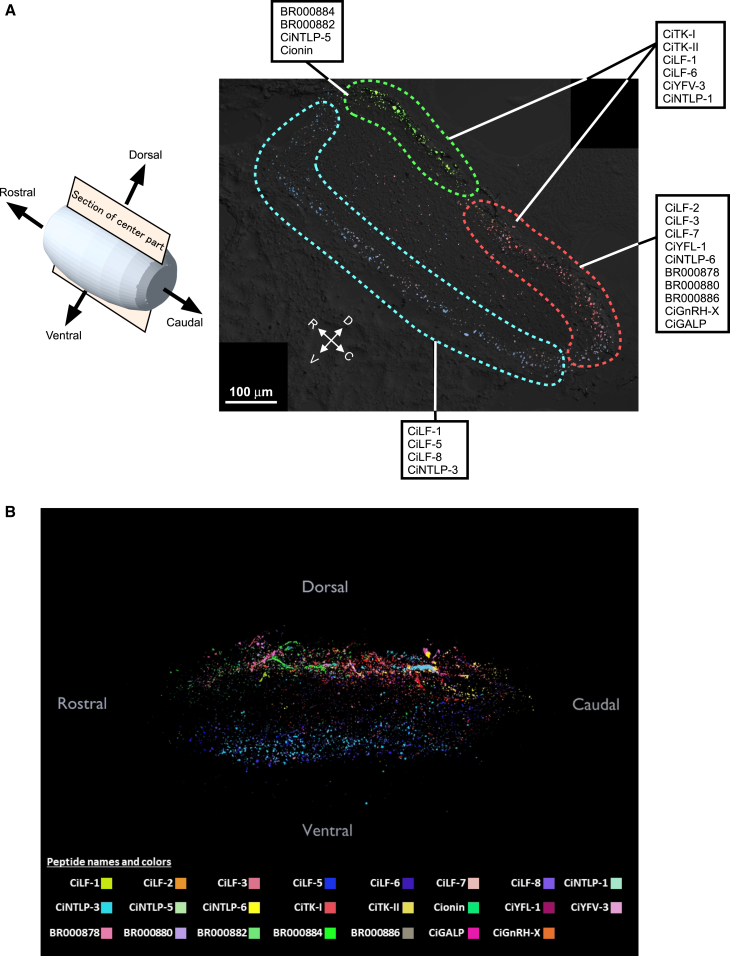
Peptide atlas of the *Ciona* cerebral ganglion (A) 2D peptide atlas of a section of the central region of the cerebral ganglion. The cerebral ganglion was divided into three regions (dotted lines) according to the distribution of the 23 peptides. The color map represents the distribution of five representative peptides (red, CiTK-I; CiLF-3, green; BR000884, CiNTLP-5, blue; Ci-LF-8). The orientation of the section is indicated by arrows. R, rostral; C, caudal; D, dorsal; V, ventral. Bar, 100 μm. (B) 3D peptide atlas of the *Ciona* cerebral ganglion. The distribution of 23 peptides is visualized in different colors. The interactive 3D atlas is available in Data S1 or in the Zenodo database as a PowerPoint file or a blender file (see key resources table). Please note that it may take a few seconds for the file to be active after opening it. See Figures S5 and S7 for the distribution of each peptide. See Figure S6 for all sections of *Ciona* cerebral ganglion divided in the three areas.

We constructed an atlas showing the distribution of *Ciona* peptides using the processed data of 36 sections covering the *Ciona* whole cerebral ganglion. In the atlas, we can visualize any combination of 23 peptides on demand. Figure 5A shows a summary of the distribution of *Ciona* peptides in sections of the central area of the cerebral ganglion. According to the distribution of the 23 peptide signals, the cerebral ganglion was divided into three areas: the dorso-rostral, dorso-caudal, and ventral areas (Figures 5A and S6). According to the 3D peptide atlas, the cerebral ganglion was also divided into three regions (Figure 5B and see key resources table). Among these areas, neuropeptides, including CiTK-I, which stimulates follicle growth in the ovary^6^^,^^12^^,^^13^^,^^14^^,^^15^ were mainly distributed in the dorso-caudal area and dorso-rostral area (Figure 5A). Interestingly, CiNTLP-6, which inhibits the effect of CiTK-I,^6^^,^^12^^,^^13^^,^^14^^,^^15^ was found to be localized in the dorso-caudal area but not in the dorso-rostral area (Figure 5A). In addition, galanin-like peptide (GALP), which participates in the regulation of reproduction in vertebrates,^39^ is also distributed in the dorso-caudal area. In contrast, cionin, which stimulates the ovulation of the follicles in the ovary,^18^ was distributed in the dorso-rostral area but not in the dorso-caudal area (Figure 5A). These results suggest that the dorsal region of the cerebral ganglion is related to the regulation of reproduction and that the functions of these ganglion components differ between the rostral and caudal regions. In the ventral area, CiLF-5, CiLF-8, and CiNTLP-3 signals were mainly localized (Figure 5A). The ventral area of the cerebral ganglion may have functions different from those of the dorsal area, although the biological functions of these peptides await further investigation.

### Colocalization and differential localization of gene-related peptides

Among *Ciona* peptides, several neuropeptide precursors produce multiple peptides (namely, gene-related peptides) that harbor similar sequences or conserved consensus motifs (Table S1); the CiLF precursor produces CiLF-1 to −8, the CiNTLP-B precursor produces CiNTLP-5 and CiNTLP-6, and the CiYFV/L precursor produces CiYFV-1 to −3 and CiYFL-1.^12^ Interestingly, the present MSI peptide atlas demonstrated that CiLF-1 was broadly distributed in the cerebral ganglion, whereas other CiLF peptides exhibited different distributions. CiLF-6 were distributed broadly in the dorsal area; CiLF-2, CiLF-3, and CiLF-7 were distributed in the dorso-caudal area; and CiLF-5 and CiLF-8 were distributed in the ventral area (Figure 5A). In addition, several neurons were found to yield all CiLFs in the dorso-caudal and ventral areas, and colocalization of CiLF-1 with other CiLFs was detected for various neurons (Figure S7A). Similarly, CiNTLP-5 and CiNTLP-6 were found to be distributed in the dorso-rostral area and dorso-caudal area, respectively, while the colocalization of the two peptide signals was observed in several neurons in the dorso-rostral area (Figures 5A and S7Ba). In contrast, the distribution of CiYFL-1 overlapped with that of CiYFV-3 in the dorso-caudal area (Figures 5A and S7Bc). Although the detection limit of MALDI-TOF MS need to be taken into account, these localization analyses suggest that the major distribution areas of several gene-related neuropeptides, encoded by their respective single precursors, show varied distribution at the neuronal level within the cerebral ganglion. Collectively, the present study revealed the detailed distribution of 23 peptides and the 3D structure of the *Ciona* cerebral ganglion.

### Colocalization of *Ciona* peptides in the cerebral ganglion

**Figure 6:**
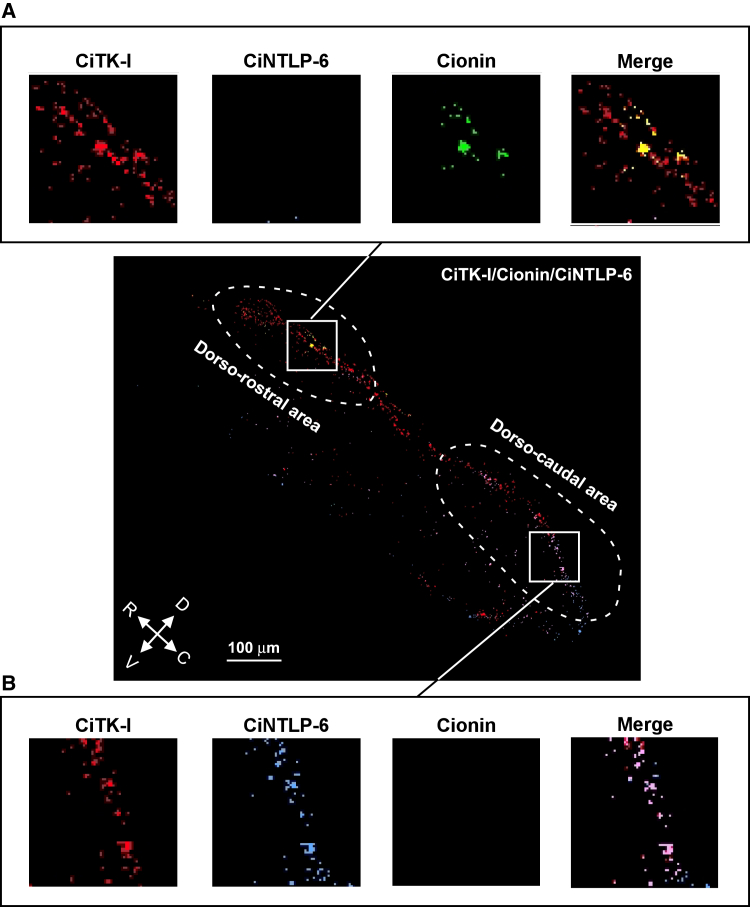
Distribution of *Ciona* reproduction-related neuropeptides in the cerebral ganglion Distribution of CiTK-I, CiNTLP-6, and cionin is shown in red, blue, and green, respectively. Rectangular areas are enlarged in (A) and (B), respectively. (A) colocalization of CiTK-I and cionin in the dorso-rostral area. (B) Colocalization of CiTK-I and CiNTLP-6 in the dorso-caudal area. The orientation of the tissue is indicated by arrows. R, rostral; C, caudal; D, dorsal; V, ventral. See Figure S7 for colocalization with other neuropeptides. Bar, 100 μm.

**Figure 7:**
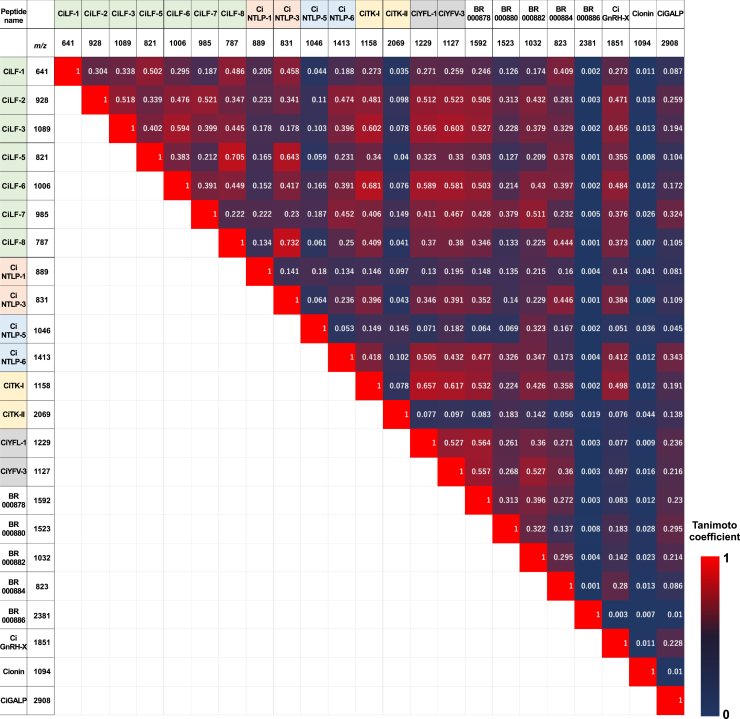
Colocalization of *Ciona* peptides Based on the Tanimoto coefficients calculated for the whole cerebral ganglion, the colocalization of each peptide was visualized as a heatmap. Peptide names and their theoretical mass values are shown. Peptides encoded by a single precursor are highlighted in the same color, whereas uncolored peptides are encoded by different precursors.

The present MSI neuronal atlas can detect and show arbitrary pairs and combinations of 23 neuropeptides on the *Ciona* cerebral ganglion, which enables the identification of various pairs or combinations of peptide colocalization at the single-cell level using the MSIs of peptides (Figure 6) and a heatmap of the Tanimoto coefficients (Figure 7). Comparison of the heatmap grouped by neuropeptide precursors demonstrated that several gene-related neuropeptides, CiYFL-1 and CiYFV-3, and CiLFs exhibited similar localization patterns, whereas CiNTLP gene-related peptides (CiNTLP-1 and CiNTLP-3, CiNTLP-5, and CiNTLP-6), and CiTK gene-related peptides (CiTK-I and CiTK-II) showed different localization (Figure 7). These results suggest the different localizations of gene-related neuropeptides or detection limit of neuropeptides as described above. CiTK-I and CiNTLP-6, which regulate follicle growth in the ovary,^6^^,^^12^^,^^13^^,^^14^^,^^15^ exhibited strong colocalization in the dorso-caudal area, but in the dorso-rostral area, CiTK-I signals were distributed in the absence of CiNTLP-6 signals (Figures 6A and 6B). Consistent with previous studies verifying that CiTK-I and CiNTLP-6 upregulate and downregulate the growth of vitellogenic follicles, respectively,^6^^,^^12^^,^^13^^,^^14^^,^^15^ these findings suggest that neurons producing both CiTK-I and CiNTLP-6 in the dorso-caudal area act as switches for stimulating and suppressing follicle growth, respectively. In the dorso-rostral region, cionin, which stimulates the ovulation of follicles,^18^ was distributed in a limited area, and cionin was almost colocalized with CiTK-I (Figures 6A and 6B). Vitellogenic follicle maturation and ovulation are sequentially regulated by these peptides in the ovary^13^^,^^15^^,^^18^; thus, these cionin/CiTK-I-producing neurons are highly likely to coordinatively regulate the biological process from vitellogenic follicle growth to ovulation. Interestingly, the peptide pairs CiTK-I/CiLF-3, CiTK-I/Ci-LF-6, and CiTK-I/CiYFL-1 exhibited high Tanimoto coefficients (>0.6), which is used to evaluate the colocalization of neuropeptides (Figure 7). Consistently, the MSI images also demonstrated that CiLF-3 and CiLF-6 are colocalized with CiTK-I and cionin in the dorso-rostral area, while CiLF-3, CiLF-6, and CiYFL-1 are colocalized with CiTK-I and CiNTLP-6 in the dorso-caudal area of the cerebral ganglion (Figure S7C). Furthermore, other peptide pairs, including CiLF-5/CiLF-8, CiLF-5/CiNTLP-3, and CiNTLP-3/CiLF-8, also exhibited high Tanimoto coefficients (>0.6) (Figure 7), and the colocalizations of these pairs were observed in the ventral area of the cerebral ganglion (Figure S7D). Taken together, the present simultaneous and efficient MSI-based neuroanatomical analysis revealed that numerous neurons in the *Ciona* cerebral ganglion unexpectedly coproduce a wide variety of multiple peptides in each area.

## Discussion

In this study, we explored 2D and 3D neuropeptidomic atlases of an invertebrate chordate CNS using a high-resolution mass spectrometry imaging method. While recent on-tissue transcriptomic approaches, including Visium and *in situ* hybridization-based high-throughput spatial transcriptomics have significantly advanced our understanding of gene expression patterns,^40^^,^^41^^,^^42^^,^^43^^,^^44^^,^^45^^,^^46^^,^^47^^,^^48^ the present neuropeptidomic spatial atlases provide new insights into the distribution of mature neuropeptides at a single-cell resolution in the *Ciona* CNS. More recently, the combination of stereo-seq and single-cell transcriptomics was used to construct a single-cell atlas of the pharyngeal organ in a non-*Ciona* ascidians.^49^ However, the construction of the comprehensive 2D map or 3D atlas has yet to be explored in the pharyngeal organ as well as the cerebral ganglion in adult *Ciona*. The most serious issue in localizing neuropeptides stems from the inability of these transcriptomic analyses to detect each gene-related peptide. This limitation arises because many neuropeptide transcripts (namely, precursors) encode multiple gene-related neuropeptides without the generation of splicing variants. Furthermore, structurally related peptides originating from the same precursors can be localized in different locations.^19^

Immunohistochemistry is a valuable method for detecting neuropeptides, including structurally related ones. However, the precise localization of individual neuropeptides using this approach can be complicated by antibody cross-reactivity, which arises from the sequence similarity among related peptides. Moreover, neuropeptides are visualized by fluorescent or chemiluminescent detection procedures, which can at most 4 neuropeptides due to the limited availability of fluorescence and chemiluminescence wavelengths in immunohistochemistry. Thus, the efficient and comprehensive localization of numerous omics-derived neuropeptides is virtually impossible by conventional immunohistochemistry. This indicates that immunostaining is insufficient for the efficient localization analysis of numerous peptides detected by omics analyses.

In this study, we explored 2D and 3D neuropeptidomic atlases of an invertebrate chordate, *Ciona robusta,* at the single-cell level using a linear interpolation-assisted MSI of neuropeptides on the peptidergic cell-visualized transgenic *Ciona*.^36^^,^^37^ These detailed anatomical analyses unveiled the distribution of numerous (twenty-three) peptides in a single section at the single-cell level without any antibodies or probes. Furthermore, structurally related peptides, such as CiLFs, CiYFLs, and CiNTLPs, which are extremely difficult to detect by immunohistochemistry due to cross-reactivity between antibodies (Figures S2A and S5A), were shown to be differentially localized (Figures S2A, S5A, and S5B). Thus, the present study successfully visualized a wide range of neuropeptides, including structurally related ones, which are still very challenging to resolve using on-tissue transcriptomics or conventional immunochemical methods. Furthermore, the utilization of linear interpolation for MSI data analyses enables the rapid and precise elucidation of spatial neuropeptide distribution throughout the entire CNS in the peptidergic cell-visualized *Ciona*^36^^,^^37^ at the single-cell level. This research procedure contrasts with previous approaches in which fluorescently labeled neurons were manually isolated and analyzed by mass spectrometry,^25^ and is expected to be applicable to other animals in which peptidergic systems have been digitally visualized. Although further improvements in quantification and high-resolution imaging along the z axis are required, the present neuropeptidomic atlases have addressed key challenges, such as single-cell level localizations of neuropeptides in whole tissues.

The cerebral ganglion of the larval *Ciona* has been compared to the vertebrate brain in relation to gene expression patterns.^50^^,^^51^ However, the functional structure of the cerebral ganglion of the adult *Ciona*, which regulates various biological functions, including reproduction,^6^^,^^13^ is more worth comparing to the vertebrate brain. The present 2D and 3D neuropeptide atlases showed that the simple oval-shaped adult *Ciona* cerebral ganglion is largely divided into at least three areas (dorso-rostral, dorso-caudal, and ventral areas) based on the distribution of twenty-three peptides (Figures 4A and 4B, and see key resources table). In the dorso-rostral area, cionin, CiNTLP-5, BR000882, and BR000884 were distributed (Figure 5A). In the dorso-caudal area, many neuropeptides, including CiLF peptides, CiYFL-1, CiNTLP-6, BR000878, BR000880, BR000886, CiGnRH-X, and CiGALP, were distributed (Figure 5A). In the ventral area, CiLF-5, CiLF-8, and CiNTLP-3 were found to be localized (Figure 5A). CiTK-I, CiTK-II, CiLF-1, CiYFV-3, and CiNTLP-1 were shown to be distributed in the dorso-rostral and dorso-caudal areas (Figure 5A). A striking feature of these peptide distributions is that several gene-related peptides (CiLFs and CiNTLPs), which are generated from the respective single precursors, exhibited differential peptide localization (Figures 5A, S7A, and S7B). These results suggest the occurrence of previously uncharacterized selective endoproteolytic processing of precursors and/or differential sorting of mature peptides in the *Ciona* cerebral ganglion, leading to the specific and diverse physiological functions of these peptides. This notion is compatible with the localization of the receptors specific to CiLFs, and CiYFV/Ls, respectively, which also suggests the specific biological roles of these neuropeptides.^52^ The investigation of biological functions of CiLFs and CiYFV/Ls will provide further insights into the structure-functional relationship of the *Ciona* cerebral ganglion. Such investigations are currently in progress. Collectively, these results also highlight the previously unrecognized functional diversification of each neuron via the differential production of gene-related peptides, which will explore the neuropeptidergic connectome of the *Ciona* cerebral ganglion.^53^

The unique localizations of the respective neuropeptides are highly likely to be associated with distinct biological roles for each of the three areas of the cerebral ganglion in the regulation of biological functions. For example, CiTK-I and CiNTLP-6, which specifically enhance and suppress the growth of late vitellogenic follicles, respectively,^6^^,^^12^^,^^13^^,^^14^^,^^15^ were observed in neurons in the dorso-caudal region (Figure 6A), whereas the ovulation inducer cionin^18^ was distributed in neurons in the dorso-rostral region (Figures 5 and 6). Additionally, our previous study using PC2-transgenic *Ciona* showed that peptidergic nerve fibers innervate the ovary.^36^^,^^37^ Collectively, these results suggest that the dorso-caudal region and the dorso-rostral region are responsible for the regulation of the growth of early-stage follicles and ovulation, respectively, in a neuropeptidergic fashion. Further investigations of the biological roles of *Ciona* neuropeptides, combined with the present neuropeptide atlases, will contribute to the exploration of the entire neuroanatomical network of the cerebral ganglion at the functional level.

The present study also verified that numerous *Ciona* cerebral neurons produce more than two types of neuropeptides, which are produced from different precursors (Figures 6 and 7). Consistent with the biological roles of CiTK-I and CiNTLP-6 as stimulators and suppressors of vitellogenic follicle growth, respectively, CiTK-I- and CiNTLP-6-coproducing neurons are highly likely to regulate both the stimulation and suppression of vitellogenic follicle growth. Similarly, CiTK-I- and cionin-coproducing neurons are thought to regulate both follicle growth and ovulation. The *Ciona* cerebral ganglion directly projects numerous peptidergic nerve fibers to various peripheral organs, including the ovary,^36^^,^^37^ suggesting that at least several of these neuropeptides are likely to be sorted and transported to different peripheral organs through distinct nerve fibers of a neuron in the *Ciona*. Altogether, the present colocalization profiles of multiple neuropeptides produced from different precursors support the view that the respective neurons are responsible for various biological functions via the secretion of multiple neuropeptides in *Ciona*.

In protostomes, the post-translational processing of the light yellow cell peptide (LYCP) precursor and the co-localization of multiple LYCPs have been demonstrated in isolated single cells of *Lymnaea* using MALDI-TOF MS.^54^ In *Aplysia*, the co-localization of egg-laying hormone and related peptides has also been investigated in isolated single bag cell neurons.^55^ In addition to the detection of multiple peptides derived from a single precursor, peptides originating from different precursors have also been found to be co-localized. In *Lymnaea*, for instance, both peptides derived from the visceral dorsal 1 (VD1) precursor and small cardioactive peptides (SCPs) derived from the SCP precursor were detected within the same neurons.^26^^,^^56^ Furthermore, neurons that produce APGWamide were also shown to produce LNPY, conopressin, or pedal peptides, each encoded by separate genes.^57^ The biological functions of the colocalized peptides originating from different precursors are thought to exert additive or subtractive effects, contributing to the fine-tuning of muscle contractions in *Lymnaea*.^57^ These findings are reminiscent of what has been observed in neurons coproducing CiTK-I/CiNTLP-6, which may have subtractive effects on vitellogenic follicle growth, and in neurons coproducing CiTK-I/cionin, which may fine-tune ovarian follicle development in the *Ciona*. Interestingly, in the dorso-rostral area, CiTK-I and cionin were further colocalized with CiLF-3 and CiLF-6, and in the dorso-caudal area, CiTK-I and CiNTLP-6 were colocalized with CiLF-3, CiLF-6, and CiYFL-1 (Figure S7C). In particular, the receptor gene for CiLF-6 is expressed specifically in the oral and atrial siphons,^52^ and these actions (opening and closing) are essential for spawning.^58^ Combined with these findings, the present results suggest that the dorsal region likely regulates not only follicle growth and ovulation but also spawning behavior, and that the dorsal region of the cerebral ganglion is the reproductive center in *Ciona*. Further investigations are needed to examine whether the present neuropeptidomic atlas exhibits biological variations consistent with physiological conditions or developmental stages with reliable experimental variations.

Compared to the case of protostomes mentioned above, in vertebrates, most neurons were shown to yield only a single peptide or with its gene-related peptides, with a few exceptions, including KNDy neurons, which produce kisspeptin, neurokinin B and dynorphin A.^59^ Therefore, the biological functions of vertebrate neurons have rarely been investigated in light of the colocalization of multiple neuropeptides. In other words, very little attention has been paid to whether neurons produce multiple neuropeptides. However, a recent single-cell transcriptome of the lateral motor cortex and primary visual cortex in mice showed that almost all neurons coexpress neuropeptide genes,^60^ suggesting that a greater number of neurons produce multiple peptides than previously expected or investigated in mammals. Combined with these findings and the phylogenetic position of *Ciona* as the closest relative to vertebrates,^2^ the present study suggests that neuropeptidergic neurons might have produced multiple neuropeptides in common ancestors of chordates as well as protostomes and that this fundamental neuronal inherence in the production of multiple neuropeptides is conserved in chordates. Further investigations of the biological roles of multiple peptide-producing neurons in *Ciona* and other vertebrates will shed new light on the functions and evolution of the central nervous system in chordates.

In conclusion, we elucidated the unexpected and useful neuropeptide landscapes in the cerebral ganglion of *Ciona*, a phylogenetically critical invertebrate chordate, using an MSI-based single-cell level neuropeptidomics. The present study verified the combination of multiple peptides produced in a single neuron and the differential production of gene-related peptides yielded from a single precursor, which cannot be verified by any transcriptome methods. The present neuropeptide atlases, integrated with the biological roles of the respective neuropeptides, also led to the exploration of relationships between regions in the cerebral ganglion and their functional roles in *Ciona* and will provide a crucial fundamental for understanding the evolution of the CNS throughout chordates. The present study also provides an efficient and powerful workflow for on-tissue single-cell level neuropeptidomics for exploring neuropeptide landscapes of various organisms.

### Limitations of this study

While this study successfully constructed two- and three-dimensional neuropeptidomic atlases of 23 peptides in the *Ciona* cerebral ganglion, several limitations remain. First, although immunohistochemical validation across different individuals showed similar distribution patterns, a broader assessment of inter-individual variability was not conducted. Second, neuropeptidomic changes under varying physiological conditions, such as reproductive stages or environmental factors, remain unexplored. Lastly, this study focused solely on neuropeptides; spatial information on low molecular weight molecules such as neurotransmitters was not included, which could further enhance our understanding of the *Ciona* nervous system.

## Resource availability

### Lead contact

Requests and further information on resources and reagents should be directed to and will be fulfilled by the Lead Contact, Honoo Satake (satake@sunbor.or.jp).

### Materials availability

The materials generated for this study are available on request from Honoo Satake (satake@sunbor.or.jp).

### Data and code availability

• Data: Original data for each Figure are available in the Mendeley Data: https://doi.org/10.17632/6679f2h3md.1. *Ciona* 3D atlas data and high-resolution supplemental Figures are also available in the Mendeley Data: https://doi.org/10.17632/8mhyj9jfn7.1 All raw Data are available from the Zenodo database (https://zenodo.org/) under the DOI numbers as shown in the key resources table. Visium data can be obtained from the NCBI Gene Expression Omnibus (GEO) database: GSE229175 (https://www.ncbi.nlm.nih.gov/geo/query/acc.cgi?acc=GSE229175).
• Code: The code for a data processing software used in this study is publicly available on GitHub: https://github.com/AS-sunbor/Imaging-MS-programs.
• Other item: Data processing software is available from the Zenodo database (https://zenodo.org/). The DOI is listed in the key resources table.
• Additional information: Any additional information required to reanalyze the data reported in this article is available from the lead contact upon request.

## Acknowledgments

We gratefully acknowledge Prof. Makoto Suematsu for his fruitful comments on the project. We thank Prof. Akira Kageyama and Dr. Naohisa Sakamoto for their fruitful discussions on image processing. We thank Dr. Daisuke Motooka for their technical support in the Visium analysis. We also thank the National Bio-Resource Project for providing ascidians. This work was supported by Grants-in-Aid for Scientific Research from the 10.13039/501100001700Ministry of Education, Culture, Sports, Science and Technology, Japan (JP22K06327 to TO, and JP22H02658 to HS).

## Author contributions

T.O., A.S., and H.S. conceived the experiments and designed the project. Methodology: T.O., A.S., K.S., T. Yamamoto, T. Yamagaki, and H.S. conceived the methodology for imaging MS analyses. Investigation: T.O., A.S., and H.S. performed experiments. T.O., A.S., and H.S. analyzed the data. Y.S. provided resources for transgenic *Ciona*. T.O., A.S., and H.S. performed a visualization of the atlas. T.O., A.S., and H.S. wrote the original draft. T.O., A.S., Y.S., K.S., T. Yamamoto, T. Yamagaki, and H.S. reviewed and edited the article. H.S. supervised the project.

## Declaration of interests

The authors declare no competing interests.

## STAR★Methods

### Key resources table

**Table:** 

REAGENT or RESOURCE	SOURCE	IDENTIFIER
**Antibodies**		

Antisera are listed in Table S2	This work	N.A.

**Deposited data**		

Interactive 3D peptide atlas data	This paper	https://zenodo.org/records/7824306
Colocalization data	This paper	https://zenodo.org/records/10278853 https://zenodo.org/records/10279156 https://zenodo.org/records/10279438
Peptide mapping data	This paper	https://zenodo.org/records/7141201
MSI raw data	This paper	https://zenodo.org/records/7020828 https://zenodo.org/records/7121589 https://zenodo.org/records/7141209 https://zenodo.org/records/7141213
Visium data	This paper	NCBI accession number: SE229175
Data processing software	This paper	https://zenodo.org/records/7297174
Code for data processing software	This paper	https://github.com/AS-sunbor/Imaging-MS-programs
Original data for each Figure	This paper	https://doi.org/10.17632/6679f2h3md.1

**Software**		

ImageJ (Fiji)	Schindelin et al.^47^	https://imagej.net/software/fiji/
GIMP	GIMP org.	https://www.gimp.org/
Blender	The Blender Foundation	https://www.blender.org/
SimlabCADVRter	Simlab soft	https://www.simlab-soft.com/

### Experimental model and subject details

*Ciona intestinalis* Type A (synonym for *Ciona robusta*)^61^ was used to create transgenic lines. *Ciona robusta* is referred to as *Ciona* in this manuscript in accordance with the description in the database of transgenic lines of the National BioResource Project of Japan (http://marinebio.nbrp.jp/). The *PC2>Kaede* line of *Ciona*, Tg[MiCiPC2K]2,^36^^,^^37^^,^^62^^,^^63^ was used for all analyses. The line was cultured using an inland culture system.^64^ Adult transgenic animals were used in this study.

### Method details

#### Tissue preparation for MSI

Animals were anesthetized using L-menthol as described previously.^36^^,^^37^ The neural complex of *Ciona* was dissected and embedded in Tissue-Tek Cryomold #4565 (Sakura Finetek Japan Co., Ltd., Tokyo, Japan) using Super Cryoembedding Medium-L1 (Leica Microsystems Japan, Tokyo, Japan). The direction of the tissues was adjusted under a SteREO Discovery stereomicroscope. V8 (Zeiss, Oberkochen, Germany). The tissues were then frozen with normal hexane cooled to -196 °C with liquid nitrogen and stored at -80 °C until sectioning was performed. The frozen tissues were serially sectioned at a thickness of 10 μm using a CryoStar NX70 cryostat (Thermo Fisher Scientific, Inc., Waltham, MA) at -18 °C. The sections were placed on indium-tin-oxide (ITO)-coated glass slides (Bruker Daltonics, Billerica, MA) and dried at room temperature. The sections were further dried in a vacuum desiccator for 5 min. A FLUOVIEW FV3000 confocal microscope with a 20× objective (Olympus, Tokyo, Japan) was used to obtain tissue images. Kaede (red) was excited at 546 nm and detected at 570-620 nm. Differential interference contrast (DIC) images of the sections were also obtained. Overview images of four glass slides containing nine tissue sections each were obtained with a Prime Histo XE slide scanner (Pacific Image Electronics Co., Ltd., New Taipei City, Taiwan) and used for positioning the tissues in the MSI system. Photoshop Elements ver.20.0 (Adobe, Inc., San Jose, CA) was used to linearly adjust the contrast of the microscope images and to tile two images for large sections. After the observation, the sections were washed in chloroform (Nacalai Tesque, Kyoto, Japan) for 20 sec to remove lipid molecules. The matrix solution (50 mg/mL 2,5-dihydroxybenzoic acid in 70% methanol and 0.1% (v/v) trifluoroacetic acid) was used to spray-coat the sections using an automated matrix sprayer (HTX TM-Sprayer; HTX Technologies, LLC., NC) with constant nitrogen flow (70 kPa). The parameters of the sprayer are as follows. Liquid flow rate: 0.1 mL/min, Nozzle velocity: 1200 mm/min, Nozzle height: 40 mm, Nozzle temperature: 60 °C, Nozzle pass time: 9 counts, Drying time: 0 sec, Track spacing: 2.5 mm.

#### Mass spectrometry imaging

MSI was performed on a rapifleX matrix-assisted laser desorption ionization time-of-flight/time-of-flight (MALDI-TOF/TOF) system (Bruker Daltonics) with a SmartBeam laser operating at 5000 Hz in positive reflectron mode. MS and MSI data acquisition were performed with FlexControl 4.0 in conjunction with flexImaging 5.0 (Bruker Daltonics). The data were acquired with a mass range of *m/z* 500-4500. The lateral resolution for MSI was set to 20 μm, and a total of 200 laser shots were accumulated per pixel at constant laser power. The ion intensities were normalized by total ion count (TIC), and two-dimensional ion intensity maps and average spectra were created by flexImaging 5.0. The mass values of the *Ciona* peptides were based on previous peptidomic data.^12^ The normalized data were exported as imzML files by flex imaging 5.0 and used for image processing. Thirty-six sections of a whole *Ciona* cerebral ganglion were analyzed. We also performed on-tissue MS/MS analysis with a single spot laser setting of 16 μm at 5000 Hz in positive lift mode. The tissue preparation was performed as described above. The data were acquired with a mass range corresponding to fragment ions from each peptide and 400 laser shots were accumulated per spot at constant laser power. A total of approximately 6,000 shots on average were accumulated for fragment ions of each peptide. Flex Analysis (v4.0) was used for the analysis of the MS/MS data, which included spectral mass adjustment, peak detection method (SNAP, signal to noise threshold: 3, maximal number of peaks: 200, SNAP average composition: Averagine), smoothing (Savitzky-Golay algorithm, width: 0.15 m/z, cycles: 4), and baseline subtraction (TopHat algorithm). The fragmentation patterns of the *Ciona* peptides were compared to those of synthetic peptides. All the MSI raw data obtained from four slides were uploaded to the Zenodo database: https://doi.org/10.5281/zenodo.7020828, https://doi.org/10.5281/zenodo.7121589, https://doi.org/10.5281/zenodo.7141209, https://doi.org/10.5281/zenodo.7141213.

#### Image processing and data integration

In this study, MSI data and fluorescence microscopy images of PC2-transgenic *Ciona*, which exhibit fluorescent signals in peptidergic cells,^36^^,^^37^ were combined to verify the distribution of peptidergic cells. First, fluorescence images obtained by microscopy were converted into 8-bit images by ImageJ (Fiji) software.^65^ Subsequently, the background of the images was subtracted (with a rolling ball radius of 25 pixels and a sliding paraboloid). The brightness/contrast was adjusted by the auto mode. The adjusted fluorescence images, differential interference contrast (DIC) images, scanned images for MSI, and exported raw MSI data (containing .imzML files and .ibd files) were input into the image processing program that was originally developed in our laboratory (Figure S1, for the software, see key resources table).^66^ The resolutions of the images were adjusted with reference to the scanned images. To align the images obtained by the confocal microscope, slide scanner, and MSI area, the x-axis, y-axis and angle of rotation values of the microscope images and the x-axis and y-axis of the MSI area were input with reference to the scanned images in the program. After reducing the backgrounds of the fluorescence images, the MSI data, fluorescence images, and DIC images were integrated by the linear interpolation method and exported as PNG files for each peptide. GIMP (https://www.gimp.org/) was used to stack all the peptide mapping images as layers in a single file. The mapping data were saved in a GIMP format, and mapping images containing arbitrary peptides were exported as PNG files to construct the peptide atlas. All the mapping data in GIMP format were uploaded to the Zenodo database: https://doi.org/10.5281/zenodo.7141201). To construct a 3D atlas, processed MSI data for 23 peptides were exported as 3D surface data (stl file) by the 3D viewer plugin of ImageJ. All the stl files were loaded into blender software (https://www.blender.org/), and pseudocolors were applied to each peptide. The 3D atlas generated with the blender software was exported as a fbx file and further converted into u3d file by SimlabCADVRter (Simlab soft). The interactive 3D atlas (Data S1) is also available as a power point file or a blender file in the Zenodo database: https://doi.org/10.5281/zenodo.7824306 (see key resources table).

#### Peptide colocalization analysis

Based on the mapping images, colocalization of the *Ciona* peptides was analyzed using ImageJ. For each peptide, the mapping images of 36 sections were loaded and combined as a stack image. The stack images were subsequently converted into 16-bit gray images. The 16-bit images were further binarized for colocalization analysis. The algorithm and threshold for binarization of the mapping images were set to “intermodes” and “8400-65535”, respectively, to optimize the background signals. To evaluate the colocalization of peptides, two sets of peptide mapping images were selected from 18 peptides, and a total of 36 images for each peptide pair were analyzed. Red and green pseudocolors were applied to the two sets of stacked images. The red stack images and green stack images were merged into a single stack image. Colocalization analysis was performed using the “Just Another Colocalization Plugin” (JACoP) BIOP version^67^ in ImageJ. In this analysis, the pixel areas of peptide “A” and peptide “B”, as well as the area of overlap were automatically counted. To evaluate colocalization, the Tanimoto coefficient^68^^,^^69^ was calculated based on the pixel area as follows.

$$Tanimoto\;coefficient=\frac{Area\;Overlap}{\left(Area\;peptide\;A\right)+\left(Area\;peptide\;B\right)-\left(Area\;Overlap\right)}$$

The Tanimoto coefficient is determined with a range of 0 to 1. A Tanimoto coefficient of 0 indicates that peptide A and peptide B are not colocalized, and a value of 1 indicates that peptide A and peptide B are completely colocalized. The Manders’ coefficients^67^^,^^69^ (M1 and M2) were also calculated to determine the ratio of the area of overlap and the area of peptide A or B as follows:

$$M1=\frac{Area\;Overlap}{Area\;peptide\;A}\;M2=\frac{Area\;Overlap}{Area\;Peptide\;B}$$

Based on the Tanimoto coefficients, colocalization of each peptide was visualized as a heatmap. Colocalized images are also visualized as tissue images. All the colocalization images are available in the Zenodo database: https://doi.org/10.5281/zenodo.10278853, https://doi.org/10.5281/zenodo.10279156, and https://doi.org/10.5281/zenodo.10279438.

#### Histochemistry

Nissl staining was performed to compare the neuronal cells and fluorescent signals. The frozen tissues of the cerebral ganglion were serially sectioned at a thickness of 10 μm as described above, and fluorescent and DIC images were obtained with a Zeiss Axio Imager. A2 (Zeiss, Oberkochen, Germany) with a red fluorescent filter (AF555). Subsequently, the sections were soaked in 4% paraformaldehyde in phosphate buffer (PB) for 60 min. The sections were washed with distilled water for 3 min and soaked in 0.1% cresyl violet acetate solution (Sigma‒Aldrich, St. Louis, MO). The stained sections were washed with 70% ethanol and 80% ethanol for 1 min each and soaked in 95% ethanol and 10% acetic acid solution for differentiation. The sections were finally dehydrated and cleared with 100% ethanol and xylene and mounted on a Mount Quick mounting medium (Daido Sangyo Co., Ltd., Tokyo, Japan). Polyclonal antibodies against CiTK-I and CiLF-2 were prepared by custom services (Hokudo Co. Ltd., Sapporo, Japan and Eurofins Genomics, Tokyo, Japan). The information for the antisera is summarized in Table S2. The specificity of all the antisera was analyzed by dot blotting as described previously (Figure S2B).^70^ The *Ciona* cerebral ganglions were fixed with Bouin’s fixative solution at 4°C overnight. The fixed tissues were soaked in 30% sucrose in PBS at 4 °C overnight. The tissues were embedded and sectioned at a thickness of 10 μm as described above. Immunoreactive products were detected with an ABC kit (Vector Laboratories, Inc., Burlingame, CA) or an NBT-BCIP kit (Nacalai Tesque, Inc., Kyoto, Japan). The specificity of the immunohistochemistry was examined using antigen-preabsorbed antisera (working dilutions, 1:1000 for the CiTK-I antiserum) with synthetic CiTK-I (50 μg/ml). Because the antiserum against CiLF-2 recognizes other structurally related CiLF peptides, including CiLF-5, CiLF-6, and CiLF-7, which are produced from a single precursor (Figure S2B),^12^ CiLF-2 immunohistochemistry was performed by preabsorbing the antiserum (working dilution, 1:8000 for the CiLF-2 antiserum) with synthetic CiLF-2, CiLF-5, CiLF-6, or CiLF-7 (50 μg/ml).

#### Visium spatial transcriptomics

Sections of the *Ciona* neural complex were prepared as described in the Tissue preparation section for MSI. Visium analysis was performed as part of contract research at the NGS Core Facility, Research Institute for Microbial Diseases, Osaka University, Japan. Before transcriptomic analysis, the total RNA quality of the tissues was analyzed via a bioanalyzer (Agilent, Santa Clara, CA), which confirmed that the RNA integrity number (RIN) was 8 or greater. Four tissue sections were mounted on a Visium Spatial Gene Expression Slide (10x Genomics, San Francisco, CA). The permeabilization time for RNA extraction was set to 15 min, and spatial transcriptomics was performed according to the manufacturer’s instructions. Transcriptome data were mapped to the *Ciona* KY21 Gene Model in *Ciona intestinalis* genomic and cDNA resources (http://ghost.zool.kyoto-u.ac.jp/download_ht.html) by Space Ranger ver. 2.0.0 (10X Genomics). The spatial gene expression was visualized by the Loupe Browser (10X Genomics). The transcriptomic data were registered in the NCBI Gene Expression Omnibus (GEO) database: GSE229175 . The orientation of the images was adjusted for comparison with the MSI results.

## Supplemental information

Document S1. Figures S1–S7, Tables S1 and S2, and Data S1

Data S2. A zip file containing raw images of Figure 6
