# Supplementary material for: Two- and three-dimensional neuropeptidomic landscape in the central nervous system of an invertebrate chordate, Ciona robusta
Source: iScience. 2025 Aug 21;28(9):113413. doi: 10.1016/j.isci.2025.113413 (PMC12441681; doi:10.1016/j.isci.2025.113413)
Supplement: Document S1. Figures S1–S7, Tables S1 and S2, and Data S1 [file mmc1.pdf]

iScience, Volume *28*

Supplemental information

Two- and three-dimensional neuropeptidomic landscape in the central nervous system of an invertebrate chordate, *Ciona robusta*

Tomohiro Osugi, Akira Shiraishi, Yasunori Sasakura, Kohtaro Sugahara, Tohru Yamagaki, Tatsuya Yamamoto, and Honoo Satake

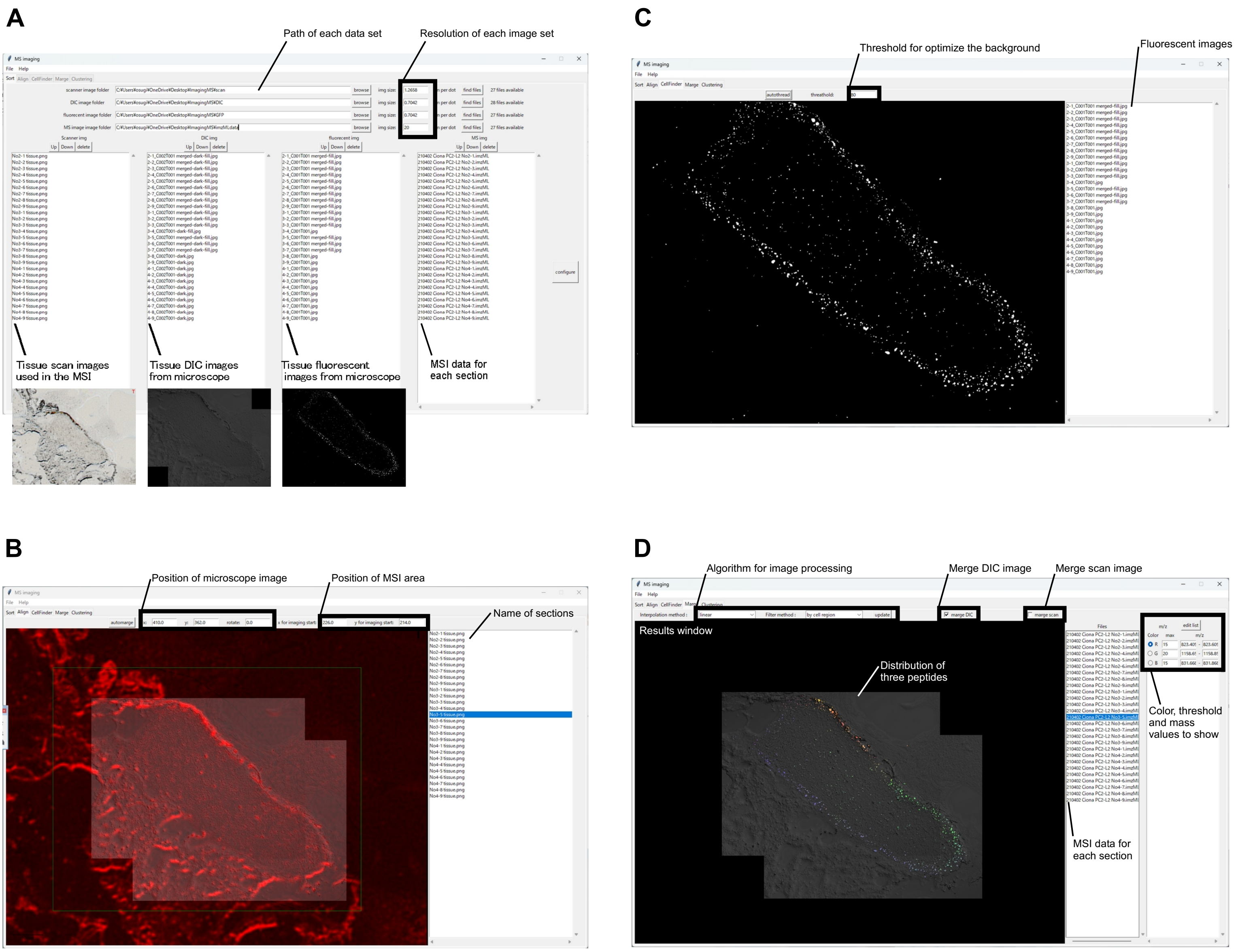

Figure S1. Screenshots of the data processing program, related to Figure 1. (A) First window for data loading and sorting. In this window, tissue scanned images, DIC images, fluorescence images and MSI data are loaded onto the program. The loaded data can be manually sorted or deleted. (B) Second window for aligning the images. To align the images obtained by the microscope, slide scanner, and MSI area, the X-axis, Y-axis and angle of rotation values of the microscope images and the X-axis and Y-axis of the MSI area were inputted with reference to the scanned images. (C) Third window for optimization of the background of fluorescence images. Input threshold to reduce the background signals of fluorescence images. (D) Fourth window for image integration. The algorithm and section are selected, and the mass values are input (± 0.2 Da for example) for visualization. Three sets of mass values are shown in red, green, and blue in the results window. This software program is available from the Zenodo database (https://zenodo.org/) with the following link https://zenodo.org/records/7297174.

(A)

(a) Fluorescent + DIC   Nissl staining

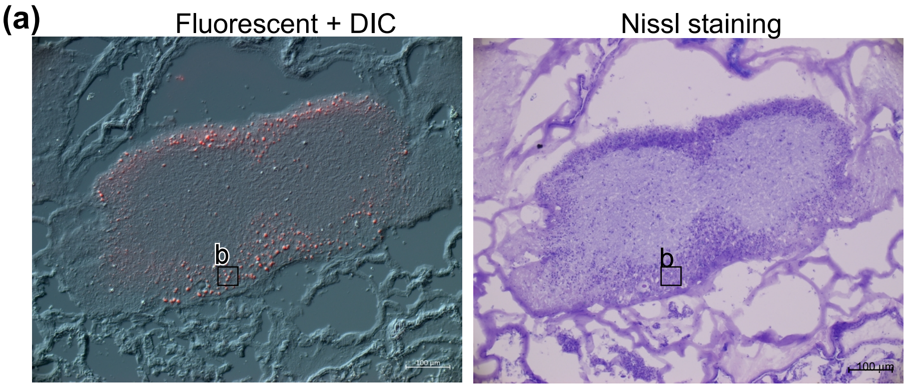

(b) Fluorescent + DIC   Nissl staining

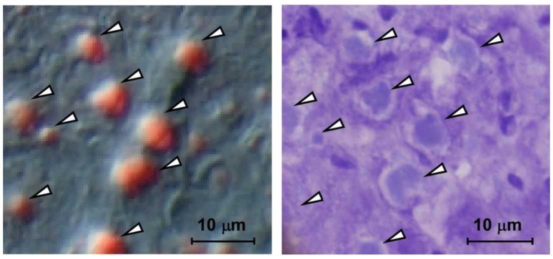

(B)

(a) Anti-CiTK-I antiserum

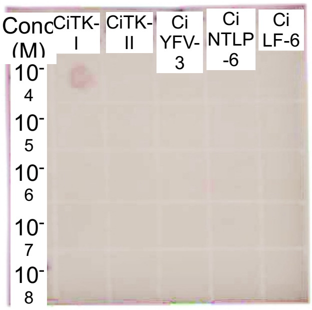

Peptides used for dot blotting

Name	Sequence
CiTK-I	HVRHFYGLMa
CiTK-II	SIGDQPSIFNERASFTGLMa
CiYFV-3	DDEPRSYFV
CiNTLP-6	SRHPKLYFPGIV
CiLF-6	SDARLQGLF

(b) Anti-CiLF-2 antiserum

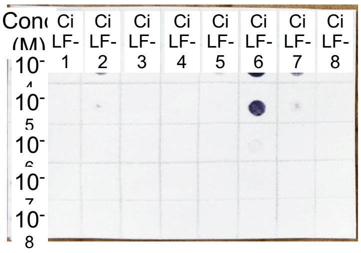

Peptides used for dot blotting

Name	Sequence
CiLF-1	FQSLF
CiLF-2	YPGFQGLF
CiLF-3	HNPHLPDLF
CiLF-4	YNSMGLF
CiLF-5	SPGMLGLF
CiLF-6	SDARLQGLF
CiLF-7	YPNFQGLF
CiLF-8	GNLHSLF

Figure S2. Histological validation of peptidergic cells and specificity of antisera used in the immunohistochemistry, related to Figures 3-6 and Figure S5A. (A) Comparison of Kaede fluorescence signals and Nissl staining in the same cerebral ganglion section of PC2 transgenic *Ciona*, which expresses Kaede among peptidergic cells. (a) Whole image of the cerebral ganglion section. (b) High magnification of the rectangular area in (a). In the same section, Kaede fluorescent signals and Nissl staining signals are shown for the same cells (arrowheads), suggesting that the Kaede-labeled cells are peptidergic neurons. (B) Dot blotting analysis of the antisera against CiTK-I and CiLF-2. (a) Antiserum against CiTK-I specifically recognizes CiTK-I. (b) Antiserum against CiLF-2 recognizes not only CiLF-2 but also CiLF-5, -6, and -7.

CiLF-8

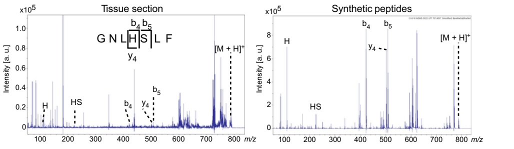

Ions	Theoretical value	Observed value (Tissue section)	Observed value (Synthetic peptide)	Error (ppm)
H	110.071	110.032	110.056	-357.0413
HS	225.098	225.054	225.072	-196.3587
b4	422.215	422.084	422.161	-309.3214
y4	503.261	503.118	503.185	-284.7427
b5	509.247	509.067	509.177	-352.8742
[M + H]+	787.410	787.248	787.264	

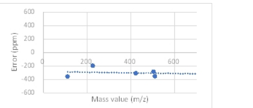

CiLF-2

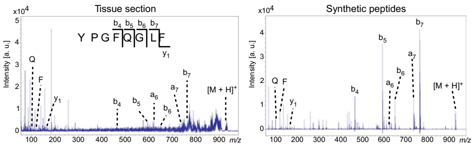

Ions	Theoretical value	Observed value (Tissue section)	Observed value (Synthetic peptide)	Error (ppm)
Q	101.071	101.049	101.053	-216.7180
F	120.081	120.048	120.069	-273.1763
y1	166.086	166.026	166.056	-363.1305
b4	465.213	465.076	465.131	-294.9707
b5	593.272	593.098	593.161	-293.0065
a6	622.298	622.089	622.205	-336.5450
b6	650.293	650.094	650.180	-306.5305
a7	735.382	735.181	735.275	-273.9111
b7	763.377	763.168	763.266	-274.3473
[M + H]+	928.456	928.220	928.329	

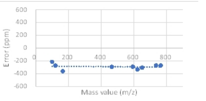

CiLF-3

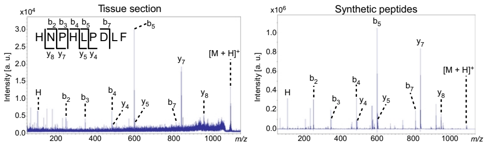

Ions	Theoretical value	Observed value (Tissue section)	Observed value (Synthetic peptide)	Error (ppm)
H	110.071	110.040	110.056	-284.4007
b2	252.109	252.045	252.062	-254.3025
b3	349.162	349.089	349.115	-208.8137
b4	486.221	486.092	486.123	-264.9535
y4	491.250	491.122	491.152	-260.6118
b5	599.305	599.141	599.187	-273.5373
y5	604.334	604.171	604.211	-269.9388
b7	811.385	811.178	811.226	-254.6763
y7	838.446	838.217	838.288	-272.9372
y8	952.489	952.223	952.325	-279.0014
[M + H]+	1089.548	1089.268	1089.405	

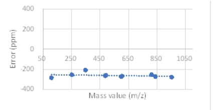

CiLF-5

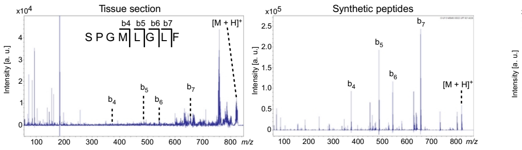

Ions	Theoretical value	Observed value (Tissue section)	Observed value (Synthetic peptide)	Error (ppm)
b4	373.154	373.065	373.096	-238.5074
b5	486.238	486.103	486.154	-277.8474
b6	543.260	543.137	543.163	-225.4908
b7	656.344	656.140	656.236	-310.2034
[M + H]+	821.423	821.278	821.276	

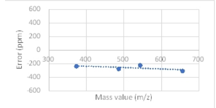

CiLF-6

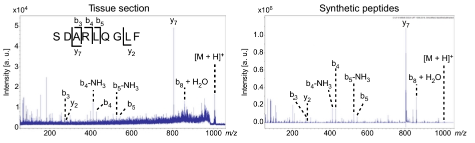

Ions	Theoretical value	Observed value (Tissue section)	Observed value (Synthetic peptide)	Error (ppm)
b3	274.103	274.035	274.075	-249.5667
y2	279.170	279.092	279.132	-280.5124
b4-NH3	413.178	413.072	413.119	-256.3426
b4	430.205	430.098	430.134	-247.5973
b5-NH3	526.262	526.103	526.172	-302.1826
b5	543.289	543.142	543.190	-269.7031
y7	804.473	804.255	804.345	-270.5307
b8+H_2O	859.463	859.205	859.334	-300.4652
[M + H]+	1006.532	1006.274	1006.305	

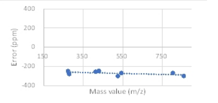

Cionin*

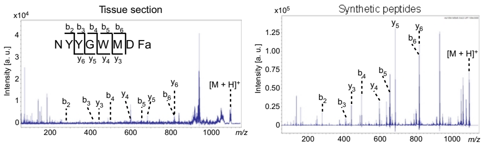

Ions	Theoretical value	Observed value (Tissue section)	Observed value (Synthetic peptide)	Error (ppm)
b2	278.114	278.084	278.086	-106.0823
y3	411.170	411.135	411.119	-84.4038
b3	441.177	441.162	441.122	-33.7775
b4	498.198	498.135	498.142	-127.0722
y4	597.249	597.149	597.180	-167.4537
y5	654.270	654.164	654.179	-162.6466
b5	684.278	684.195	684.190	-120.7267
b6	815.318	815.218	815.210	-122.7904
y6	817.334	817.194	817.217	-171.0684
[M + H]+	1094.440	1094.350	1094.261	

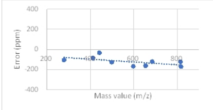

Ci-YFV-3

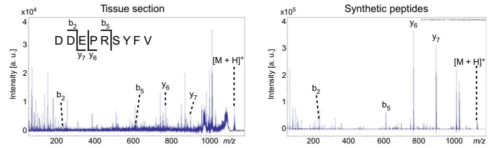

Ions	Theoretical value	Observed value (Tissue section)	Observed value (Synthetic peptide)	Error (ppm)
b2	231.061	231.038	231.038	-100.4063
b5	613.258	613.131	613.144	-206.4385
y6	768.404	768.202	768.270	-262.7524
y7	897.447	897.198	897.299	-276.8967
[M + H]+	1127.500	1127.331	1127.362	

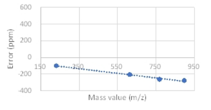

CiYFL-1

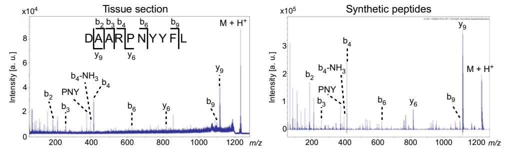

Ions	Theoretical value	Observed value (Tissue section)	Observed value (Synthetic peptide)	Error (ppm)
b2	187.071	187.025	187.069	-247.5023
b3	258.108	258.025	258.092	-323.1406
PNY	375.166	375.071	375.128	-254.0466
b4-NH3	397.183	397.080	397.145	-259.3511
b4	414.210	414.098	414.157	-269.4630
b6	625.305	625.152	625.234	-245.0283
y6	816.393	816.206	816.300	-228.7149
b9	1098.500	1098.236	1089.404	-242.6097
y9	1114.568	1114.287	1114.463	-252.1394
[M + H]+	1229.595	1229.309	1229.538	

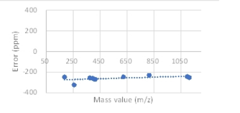

CiGnRH-X

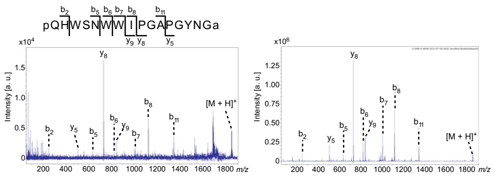

Ions	Theoretical value	Observed value (Tissue section)	Observed value (Synthetic peptide)	Error (ppm)
b2	249.098	249.042	249.064	-225.6448
y5	506.236	506.125	506.156	-218.9048
b5	636.253	636.166	636.187	-135.9663
y8	731.347	731.205	731.281	-194.3165
b6	822.332	822.200	822.258	-160.2903
y9	844.431	844.282	844.364	-176.7010
b7	1008.411	1008.187	1008.306	-222.2540
b8	1121.495	1121.249	1121.368	-219.5533
b11	1346.607	1346.309	1346.419	-220.9565
[M + H]+	1851.835	1851.512	1851.517	

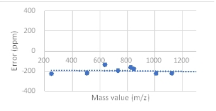

CiTK-I

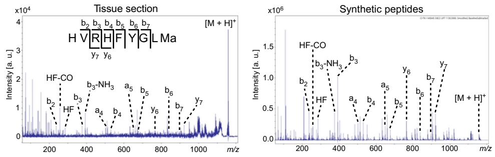

Ions	Theoretical value	Observed value (Tissue section)	Observed value (Synthetic peptide)	Error (ppm)
b2	237.135	237.057	237.111	-327.2729
HF-CO	257.140	257.064	257.125	-294.4093
HF	285.135	285.050	285.106	-296.7317
b3-NH3	376.209	376.090	376.170	-316.8780
b3	393.236	393.106	393.193	-329.8635
a4	502.300	502.163	502.282	-272.1579
b4	530.295	530.115	530.240	-338.7145
a5	649.368	649.134	649.333	-360.5238
b5	677.363	677.125	677.290	-351.4004
y6	766.371	766.128	766.286	-316.4615
b6	840.426	840.099	840.332	-389.6079
b7	897.448	897.159	897.365	-321.8311
y7	922.472	922.154	922.377	-344.3278
[M + H]+	1158.599	1158.273	1158.531	

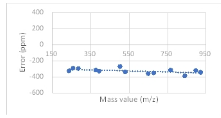

CiNTLP-6

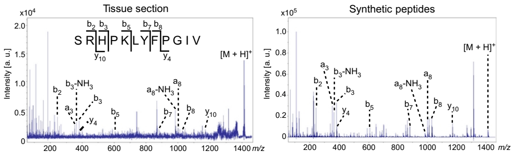

Ions	Theoretical value	Observed value (Tissue section)	Observed value (Synthetic peptide)	Error (ppm)
b2	244.140	244.066	244.103	-304.7894
a3	353.204	353.136	353.166	-193.6766
b3-NH3	364.173	364.080	364.116	-254.8638
b3	381.199	381.097	381.126	-268.4152
y4	385.245	385.138	385.179	-276.4948
b5	606.347	606.193	606.256	-254.1831
b7	882.494	882.270	882.351	-254.3206
a8-NH3	984.541	984.166	984.385	-381.3549
a8	1001.568	1001.300	1001.437	-267.5156
b8	1029.563	1029.315	1029.390	-240.8222
y10	1170.667	1170.272	1170.472	-337.4707
[M + H]+	1413.800	1413.518	1413.646	

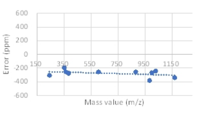

BR000882

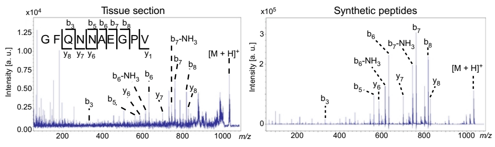

Ions	Theoretical value	Observed value (Tissue section)	Observed value (Synthetic peptide)	Error (ppm)
b3	333.156	333.078	333.102	-233.2243
b5	561.242	561.098	561.124	-255.8613
y6	586.283	586.105	586.160	-303.7782
b6-NH3	615.252	615.092	615.123	-260.3810
b6	632.279	632.107	632.145	-271.5675
y7	700.326	700.121	700.177	-292.7208
b7-NH3	744.295	744.080	744.142	-288.4610
b7	761.321	761.119	761.163	-265.7222
b8	818.343	818.121	818.179	-271.0856
y8	828.385	828.184	828.221	-242.1581
[M + H]+	1032.475	1032.245	1032.270	

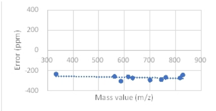

BR000878

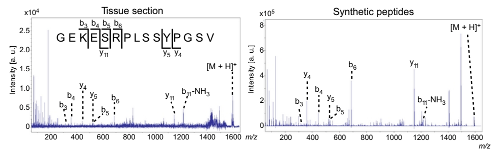

Ions	Theoretical value	Observed value (Tissue section)	Observed value (Synthetic peptide)	Error (ppm)
b3	315.166	315.048	315.048	-375.4983
y4	359.193	359.077	359.077	-321.6580
b4	444.209	444.084	444.130	-281.7240
y5	522.256	522.147	522.159	-208.3657
b5	531.241	531.111	531.140	-244.5683
b6	687.342	687.203	687.236	-202.2595
y11	1149.590	1149.270	1149.363	-278.3281
b11-NH3	1217.580	1217.222	1217.222	-293.8659
[M + H]+	1592.791	1592.496	1592.562	

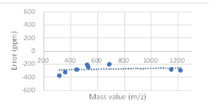

Figure S3. MS/MS spectra of native peptides from cerebral sections and synthetic peptides (above two panels for each peptide), related to Figures 2 and 5. The X- and Y-axes show the mass (m/z) and intensity, respectively. Fragment ions, including b-ions and y-ions, are indicated in each spectrum. The amino acid sequence and detected fragment ions are also indicated in each spectrum. Mass values of each fragment ion are summarized (left bottom panel). Graphs at the right bottom panel show mass errors of the fragment ions detected in cerebral sections compared to the theoretical values. *, nonsulfated form.

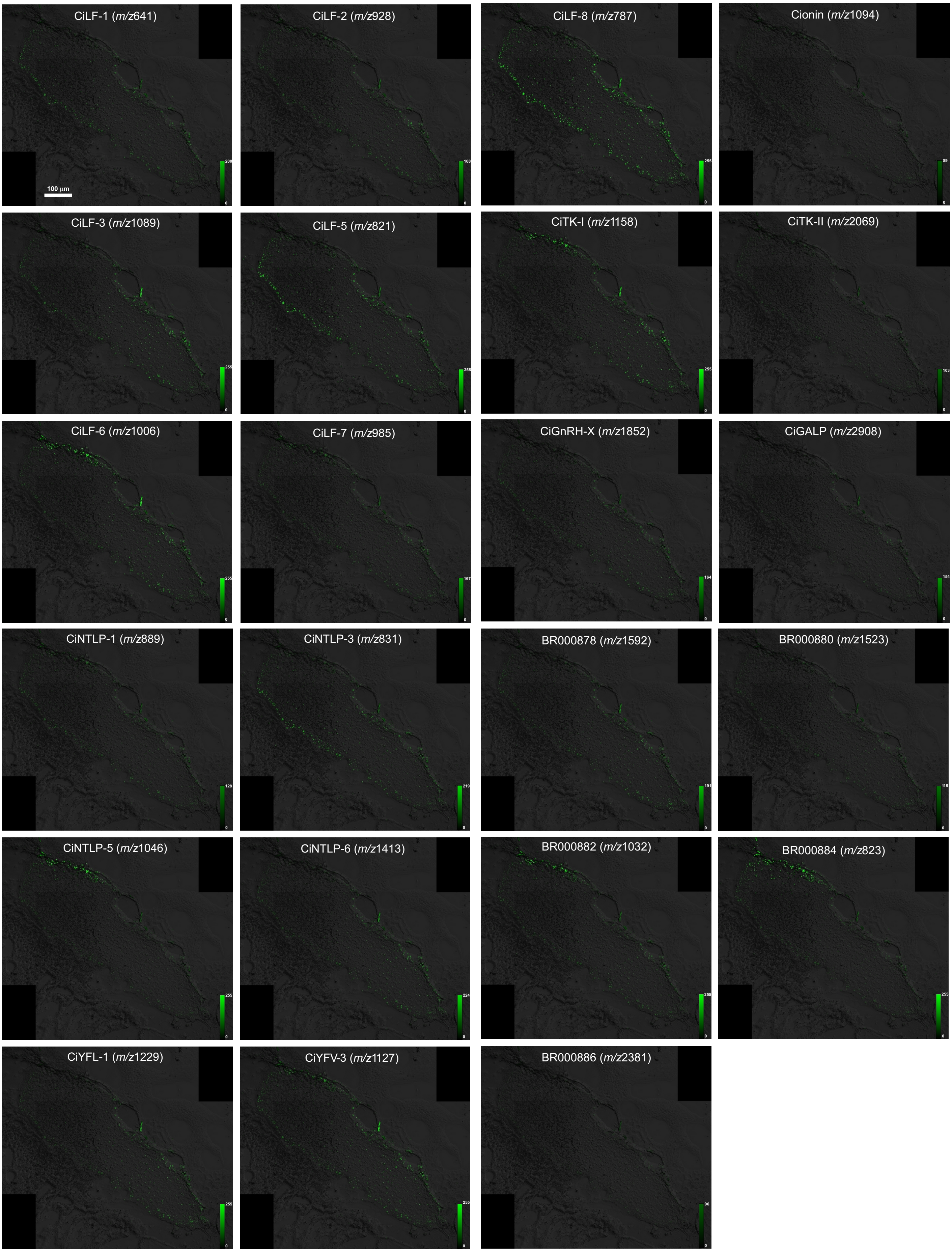

Figure S4. Processed MSI data of 23 peptides in a single section, related to Figures 3-5. The distribution of neuropeptides is shown in green. Peptide names and mass values are indicated in each image. Note that the signal of BR000886 is very weak in this section.

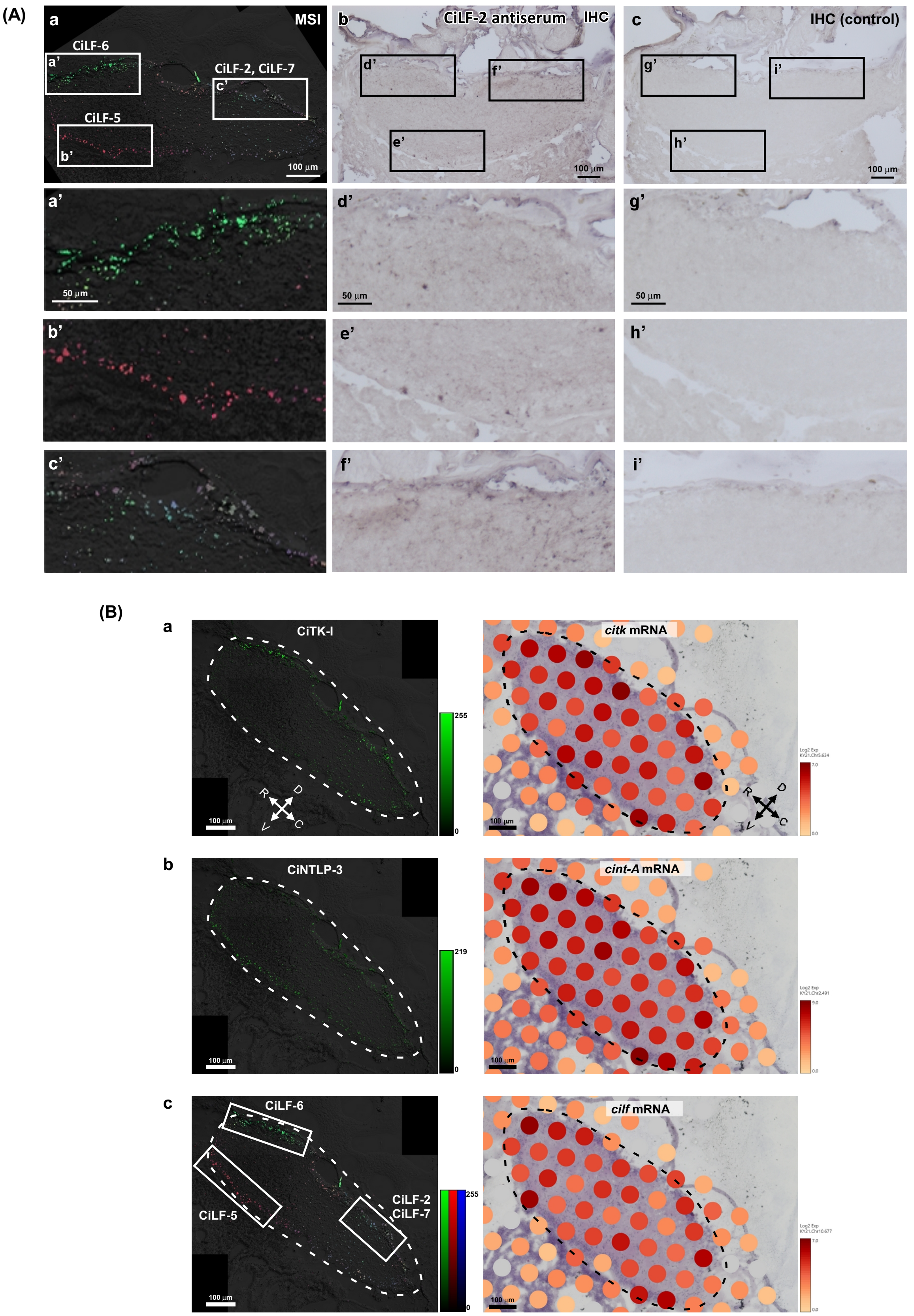

Figure S5. Comparison between the MSI and histochemistry for cerebral ganglion sections, related to Figure 5. (Aa) MS image of CiLF-2, CiLF-5, CiLF-6, and CiLF-7 in the cerebral ganglion. The rectangular areas (a' : CiLF-2, b' : CiLF-5, c' : CiLF-2 and CiLF-7) where the peptides are mainly localized are magnified below. Pseudocolor was applied for CiLF-6 (green), CiLF-5 (red), and Ci-LF-2 and CiLF-7 (blue). (Ab) Immunohistochemistry using antiserum against CiLF-2 in the cerebral ganglion. The rectangular areas (d' -f') where immunoreactive signals were distributed are magnified below. (Ac) Negative control for immunohistochemistry using antiserum preabsorbed with CiLF-2, CiLF-5, CiLF-6, or CiLF-7. The rectangular areas (g' -I') are magnified below. All the immunoreactivities disappeared in the control group. See Figure S2 for the specificity of the antiserum against CiLF-2. (Ba) Distribution of the CiTK-I and citk mRNA (right). (Bb) Distribution of the CiNTLP-3 and cint-A mRNA. (Bc) Distribution of the CiLF-2, -5, -6, and -7 and the cilf mRNA. The cerebral ganglion is indicated by a dotted line. The color bar in the MSI image indicates the brightness intensity of the pixels. The color bar in the Visium indicates the intensity of gene expression. The orientation of the section is indicated by arrows. D, dorsal; V, ventral; R, rostral; C, caudal.

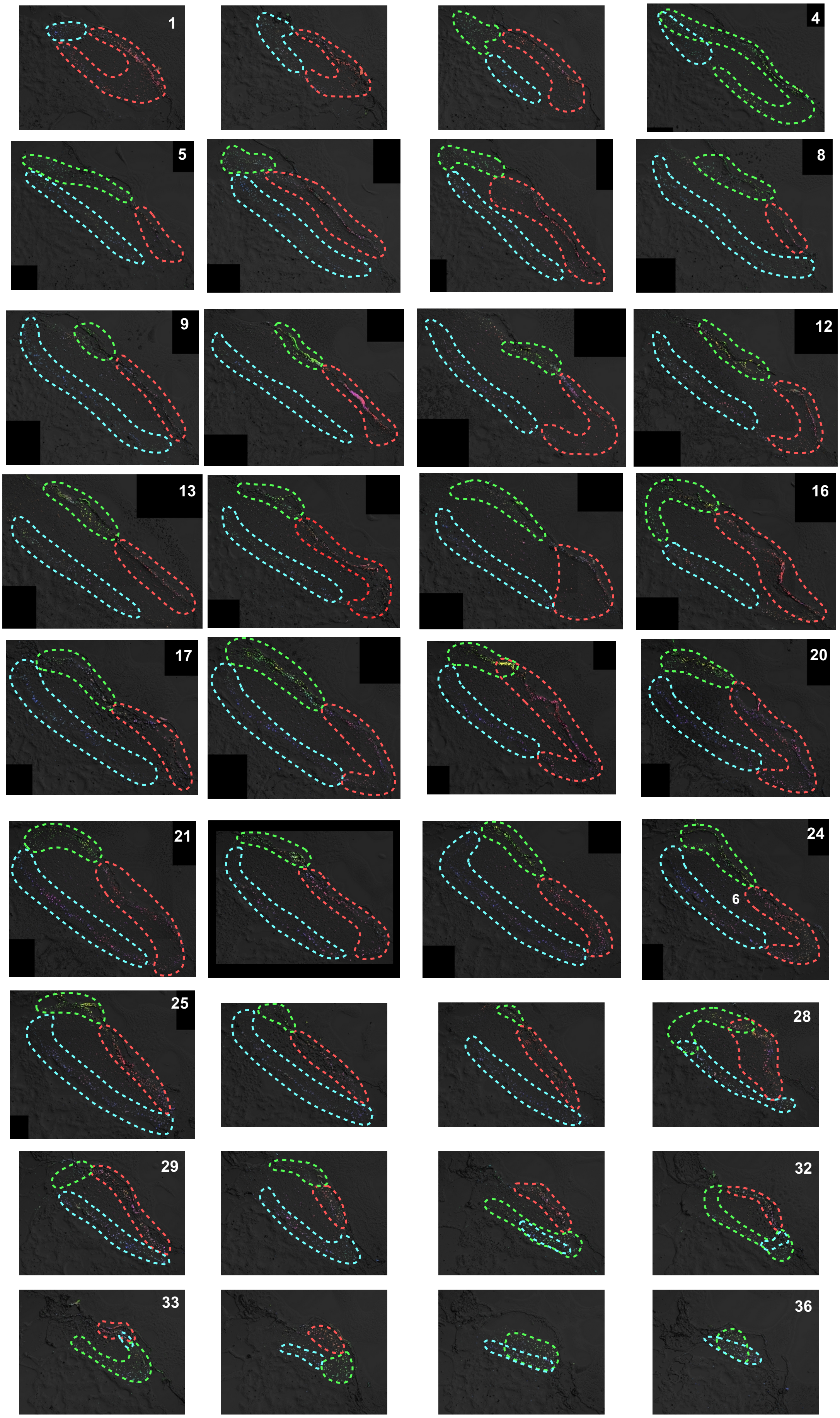

Figure S6. The *Ciona* cerebral ganglion can be divided into three areas based on the peptide distribution, related to Figure 5. The images show serial sections of the whole cerebral ganglion. The color map represents the distribution of five representative peptides (red, CiTK-I; CiLF-3, green; BR000884; CiNTLP-5, blue; CiLF-8). The three areas are indicated by broken lines. The number of sections is indicated on both sides.

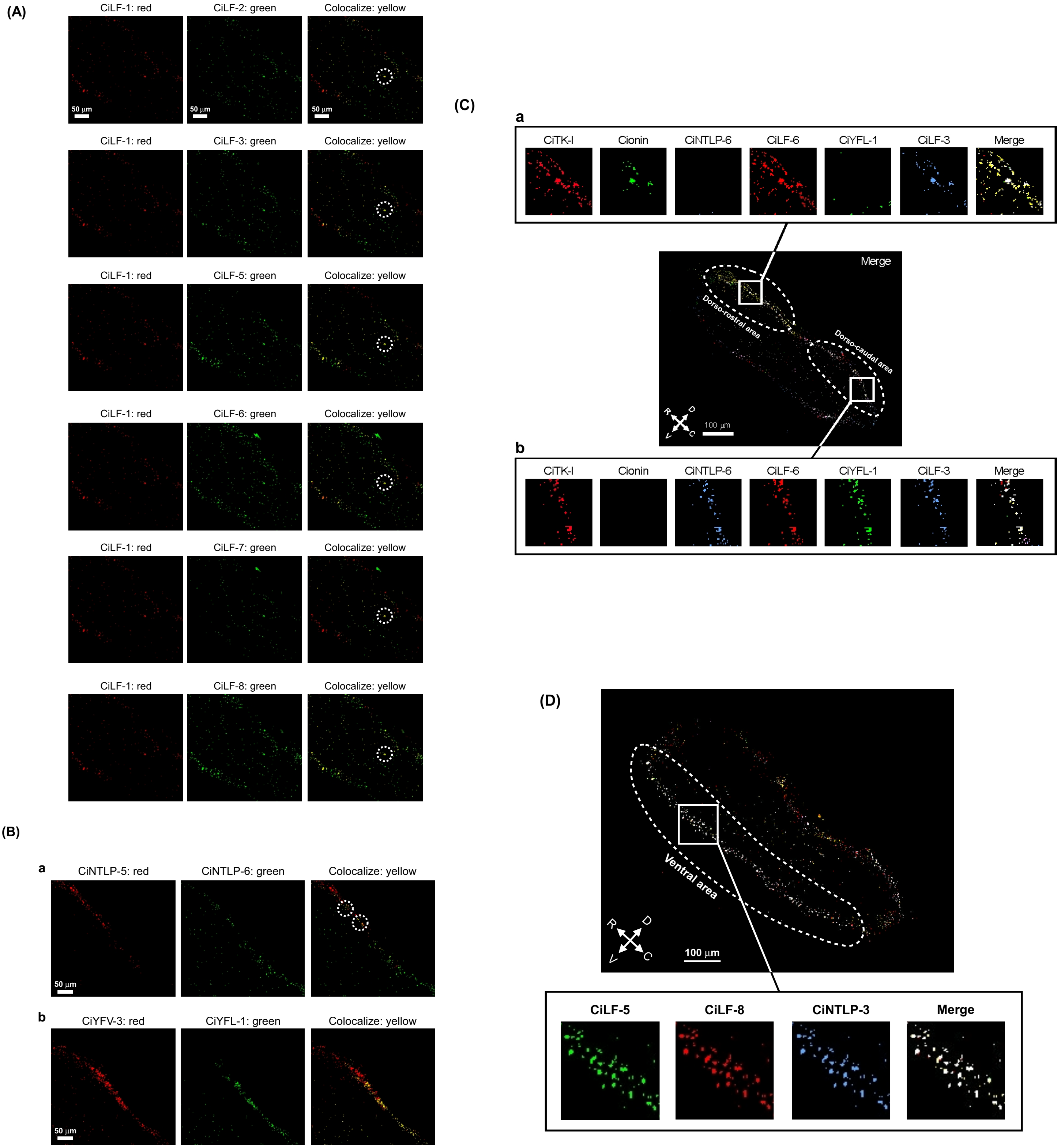

Figure S7. Colocalization of Ciona neuropeptides in various areas, related to Figure 5. (A) CiLF peptides are produced from a single precursor protein but exhibit various localizations. The dotted circle shows a cell in which all the CiLF peptides were localized within a single cell. (Ba) CiNTLP-5 and CiNTLP-6, which are produced from a single precursor protein, exhibited largely different localizations. The dotted circle shows a cell in which CiNTLP-5 and CiNTLP-6 were localized in a single cell. (Bb) CiYFV-3 and CiYFL-1 were produced from a single precursor protein, and the localization of CiYFL-1 overlapped with that of CiYFV-3. Scale bars = 50 μm. (C) Colocalization of reproduction-related neuropeptides (CiTK-I, CiNTLP-6, and cionin) and other neuropeptides (CiLF-6, CiYFL-1, and CiLF-3) in the cerebral ganglion. Middle panel shows a whole image of the tissue section. Rectangular areas are enlarged in (Ca) and (Cb). (Ca) Colocalization of CiTK-I, CiLF-6, cionin, CiLF-6, CiYFL-1, and CiLF-3 in the dorso-rostral area. (Cb) Colocalization of CiTK-I, CiLF-6, cionin, CiLF-6, CiYFL-1, and CiLF-3 in the dorso-caudal area. (D) Colocalization of CiLF-5, CiLF-8, and CiNTLP-3 in the ventral area of the cerebral ganglion. The upper panel shows a whole image of the cerebral ganglion. The rectangular area is enlarged in the lower panels, and the distribution of CiLF-5, CiLF-8, CiNTLP-3, and merged images are shown. The orientation of the section is indicated by arrows. D, dorsal; V, ventral; R, rostral; C, caudal.

Table S1. Type or paste table title here. Paste table below the title, related to Figures 2-6.

Gene name (Gene ID)	Peptide name	Peptide sequence	Theoretical mass value ([M + H]$^+$)
cilf (KY.Chr10.936.v1.ND1-1)	CiLF-1	FQSLF	641.33
	CiLF-2	YPGFQGLF	928.46
	CiLF-3	HNPHLPDLF	1089.55
	CiLF-5	SPGMLGLF	821.42
	CiLF-6	SDARLQGLF	1006.53
	CiLF-7	YPNFQGLF	985.48
	CiLF-8	GNLHSLF	787.41
citk (KY.Chr5.628.v1.SL3-1)	CiTK-I	HVRHFYGLMa	1158.59
	CiTK-II	SIGDQPSIFNERASFTGLMa	2069.01
cint-A (KY.Chr2.889.v2.nonSL11-1)	CiNTLP-1	pQLHVPSIL	889.51
	CiNTLP-3	MMLGPGIL	831.45
cint-B (KY.Chr2.888.v1.SL1-1)	CiNTLP-5	NKLLYPSVI	1046.62
	CiNTLP-6	SRHPKLYFPGIV	1413.80
ciyfv/l (KY.Chr2.2142.v1.SL1-1)	CiYFV-3	DDEPRSYFV	1127.50
	CiYFL-1	DAARPNYYFL	1229.59
cigalp (KY.Chr4.1062.v1.nonSL6-1)	CiGALP	PFRGQGGWTLNSVGYNAGLGALRKLFE	2908.52
cignrh-x (KY.Chr5.955.v1.SL2-1)	CiGnRH-X	pQHWSNWWIPGAPGYNGa	1851.84
cionin (KY21.Chr9.654.v1.SL1-1)	Cionin*	NYYGWMDFa	1094.44
n/a (KY21.Chr7.1153.v1.SL1-1)	BR000884	WLRYDA	823.41
n/a (KY21.Chr1.1164.v1.SL1-1)	BR000882	GFQNNAEGPV	1032.47
n/a (KY21.Chr12.988.v1.SL1-1)	BR000880	AVLHLAINEFQRL	1523.87
n/a (KY21.Chr4.129.v1.SL1-1)	BR000878	GEKESRPLSSYPGSV	1592.79
n/a (KY21.Chr4.132.v1.SL1-1)	BR000886	NLLSLLQHAIETANNAYRSPR	2381.27

pQ and "a" denote a pyroglutamin residue and the C-terminal amidation. *, nonsulfated form. *n/a*, gene name is not determined and not applicable. Gene IDs are indicated in parentheses.

Table S2. Information of custom antisera against CiTK-I, and CiLF-2, related to Figure 4 and Figure S6.

Name	Animal	Antigen peptide	Supplier
CiTK-I antiserum	Mouse	CHVRHFYGLM-NH_2	Hokudo Co. Ltd., Sapporo, Japan
CiLF-2 antiserum	Rabbit	CYPGFQGLF	Eurofins Genomics, Tokyo, Japan

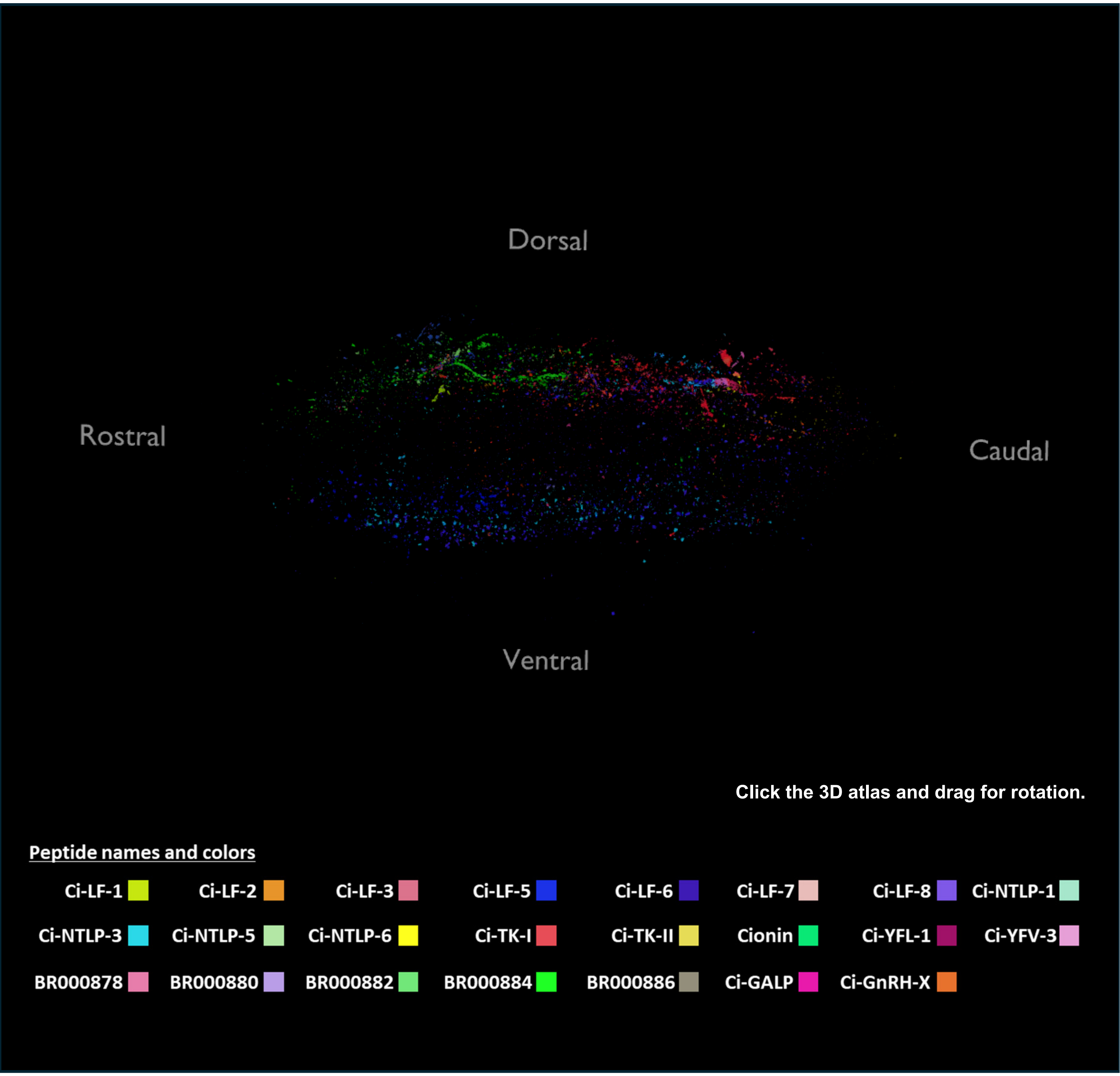

Data S1. 3D interactive peptide map of the *Ciona* cerebral ganglion.
