# Supplementary figures and images for: Two- and three-dimensional neuropeptidomic landscape in the central nervous system of an invertebrate chordate, Ciona robusta
Source: iScience. 2025 Aug 21;28(9):113413. doi: 10.1016/j.isci.2025.113413 (PMC12441681; doi:10.1016/j.isci.2025.113413)

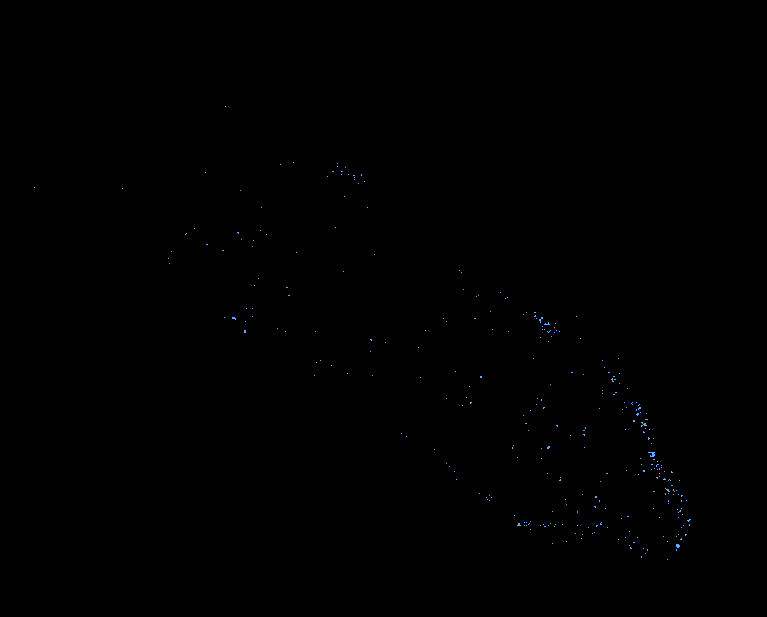

Supplement: Data S2. A zip file containing raw images of Figure 6 [file mmc2.zip › CiNTLP-6 blue.png]

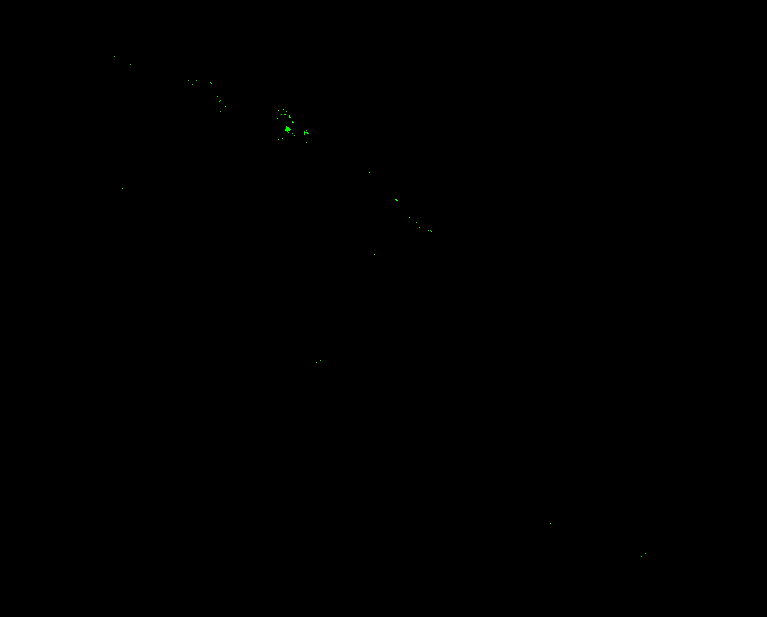

Supplement: Data S2. A zip file containing raw images of Figure 6 [file mmc2.zip › Cionin-green.png]

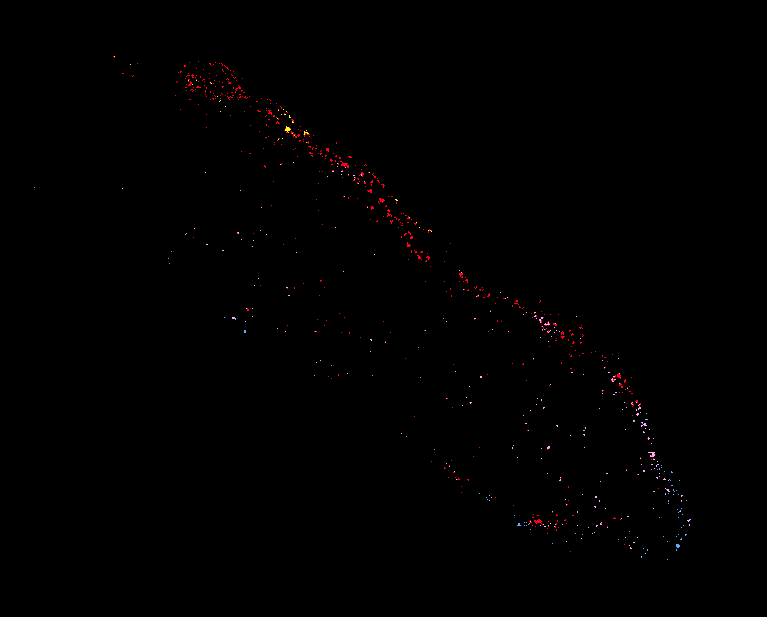

Supplement: Data S2. A zip file containing raw images of Figure 6 [file mmc2.zip › CiTK-CiNTLP-6-Cionin-merged.png]

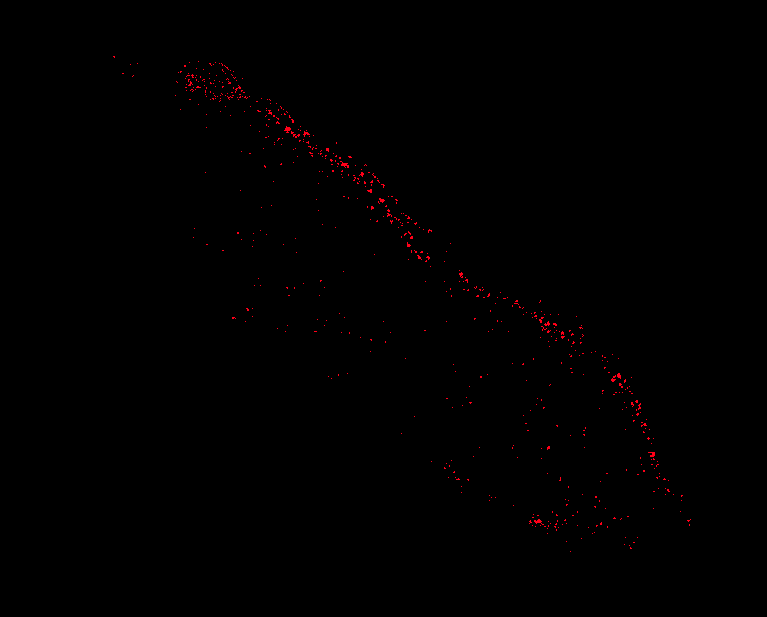

Supplement: Data S2. A zip file containing raw images of Figure 6 [file mmc2.zip › CiTK-red.png]
